# How effective are social norms interventions in changing the clinical behaviours of healthcare workers? A systematic review and meta-analysis

**DOI:** 10.1186/s13012-020-01072-1

**Published:** 2021-01-07

**Authors:** Mei Yee Tang, Sarah Rhodes, Rachael Powell, Laura McGowan, Elizabeth Howarth, Benjamin Brown, Sarah Cotterill

**Affiliations:** 1 Centre for Biostatistics, Division of Population Health, Health Services Research and Primary Care, School of Health Sciences, Faculty of Biology Medicine and Health, University of Manchester, Oxford Road, Manchester, M13 9PL UK; 2 National Institute of Health Research Behavioural Science Policy Research Unit, Population Health Sciences, Baddiley-Clark Building, Faculty of Medical Sciences, Newcastle University, Newcastle Upon Tyne, NE2 4AX UK; 3 Manchester Centre for Health Psychology, Division of Psychology and Mental Health, School of Health Sciences, Faculty of Biology Medicine and Health, University of Manchester, Manchester, M13 9PL UK; 4 Health e-Research Centre, Farr Institute for Health Informatics Research, Faculty of Biology Medicine and Health, University of Manchester, Manchester, M13 9PL UK; 5 Centre for Primary Care, School of Health Sciences, Faculty of Biology Medicine and Health, University of Manchester, Manchester, M13 9PL UK

**Keywords:** Systematic review, Meta-analysis, Health professional behaviour, Social norm, Social comparison, Information about others’ approval, Credible source, Social reward, Social incentive, Audit and feedback

## Abstract

**Background:**

Healthcare workers perform clinical behaviours which impact on patient diagnoses, care, treatment and recovery. Some methods of supporting healthcare workers in changing their behaviour make use of social norms by exposing healthcare workers to the beliefs, values, attitudes or behaviours of a reference group or person. This review aimed to evaluate evidence on (i) the effect of social norms interventions on healthcare worker clinical behaviour change and (ii) the contexts, modes of delivery and behaviour change techniques (BCTs) associated with effectiveness.

**Methods:**

Systematic review and meta-analysis of randomised controlled trials. Searches were undertaken in seven databases. The primary outcome was compliance with a desired healthcare worker clinical behaviour and the secondary outcome was patient health outcomes. Outcomes were converted into standardised mean differences (SMDs). We performed meta-analyses and presented forest plots, stratified by five social norms BCTs (*social comparison*, *credible source*, *social reward*, *social incentive* and *information about others’ approval*). Sources of variation in social norms BCTs, context and mode of delivery were explored using forest plots, meta-regression and network meta-analysis.

**Results:**

Combined data from 116 trials suggested that social norms interventions were associated with an improvement in healthcare worker clinical behaviour outcomes of 0.08 SMDs (95%CI 0.07 to 0.10) (*n* = 100 comparisons), and an improvement in patient health outcomes of 0.17 SMDs (95%CI 0.14 to 0.20) (*n* = 14), on average. Heterogeneity was high, with an overall *I*^2^ of 85.4% (healthcare worker clinical behaviour) and 91.5% (patient health outcomes). *Credible source* was more effective on average, compared to control conditions (SMD 0.30, 95%CI 0.13 to 0.47, *n* = 7). *Social comparison* also appeared effective, both on its own (SMD 0.05, 95%CI 0.03 to 0.08, *n* = 33) and with other BCTs, and seemed particularly effective when combined with *prompts/cues* (0.33, 95%CI 0.22 to 0.44, *n* = 5).

**Conclusions:**

Social norms interventions appeared to be an effective method of changing the clinical behaviour of healthcare workers and have a positive effect on patient health outcomes in a variety of health service contexts. Although the overall result is modest and variable, there is the potential for social norms interventions to be applied at large scale.

**Trial registration:**

PROSPERO CRD42016045718.

Contributions to the literature

- This is the first systematic review and meta-analysis on the use of social norms interventions to change the clinical behaviour of healthcare workers, and the results suggest that, on average, these interventions are effective.
- Social norms interventions may be effective across a range of health service contexts and modes of delivery, but the effects are variable.
- These findings contribute to a recognised gap in the literature, by highlighting which social norms interventions may be most effective: this can inform the design of future interventions aimed at improving health professional practice.

## Background

Healthcare workers routinely perform behaviours in clinical settings which impact all aspects of patient care including diagnoses, treatment and recovery. There are best-practice guidelines for many of these clinical behaviours. For example, regular blood glucose testing for diabetic patients. Healthcare workers face many challenges in following evidence-based professional practice such as lack of time, competing demands and requests from patients. Although there are no reliable published estimates of how well healthcare workers follow best clinical practices, 1 in 20 hospital admissions is caused by adverse drug events [1], and approximately half of these globally are believed to be due to lapses in best practice in terms of prescribing or monitoring behaviours by clinicians [2].

Social influences are important in clinical practice: prescribers of antibiotics have reported that pressure from patients and other prescribers in their networks influence their prescribing behaviours [3]. Social norms can be broadly considered as the perceived implicit or explicit behavioural rules that one uses to determine the appropriate and/or typical expectations, beliefs, attitudes and behaviours of a social reference person or group [4]. We have defined a social norms intervention as one which seeks to change the clinical behaviour of a target healthcare worker by exposing them to the values, beliefs, attitudes or behaviours of a reference group or person. The target healthcare worker is the person at whom a social norms intervention is aimed, with a view to changing their clinical behaviour. The reference person or group describes a person or group whose values, beliefs or behaviours are exposed to the target. Social norms interventions sometimes report a peer benchmark, such as the top 10% of the reference group or the average performance: the downside of the average approach is that the above-average performers will receive feedback suggesting that they are already performing better than their peers, and this may lead them to reduce their effort [5].

Behaviour change interventions based on social norms may help overcome barriers to healthcare workers implementing recommended practice through: persuasion, encouraging collaboration to achieve change, observing good practice from elsewhere and support from management [6]. There are various explanations of the processes through which social norms impact on behaviour according to social and health psychology theories. Social comparison theory [7] proposes that individuals draw on social comparisons to evaluate one’s abilities and perform behaviours which will bring one's abilities in line with those of others in the group. According to the social identity perspective [8], people make evaluations about their own group (‘in group norms’) against other groups (‘out group norms’). They are motivated to preserve their social identity (as part of their ‘in group’) by behaving in similar ways to the group’s normative behaviour. ‘Subjective norm’ is a construct within the Theory of Planned Behaviour [9], which describes an individual’s perception of whether valued others think they should perform a behaviour, combined with a motivation to comply with others’ beliefs.

A social norms intervention with a descriptive norms [10] message provides the target with information about the behaviour of others in the reference group (such as providing a nurse with information about the behaviours of nurses regarding wound dressing). An injunctive norms message provides the target worker with information about the values, beliefs or attitudes of the reference group towards a particular behaviour, conveying social approval or disapproval (e.g. saying that colleagues disapprove of ordering unnecessary tests). This includes approval, praise, commendation, applause or thanks.

Audit and feedback (A&F) is a quality improvement technique used by health services, where data is collected on healthcare worker performance and then a summary is reported back to the individual [11]. Social norms interventions are sometimes included as one component of A&F, usually by providing descriptive norms of others’ behaviour [12, 13]. A&F has already been shown to be effective in changing healthcare worker behaviour, but with large variation in outcomes depending on the context and the intervention design [14]. There is a need to understand the ingredients for successful A&F [11, 15], and the effects or mechanisms of the ‘social influence’ constituents of A&F have been identified as topics for further research [11]. Our review contributes to this important research agenda by systematically examining the evidence for using social norms interventions with healthcare workers.

Identification of the individual components within social norms interventions can aid understanding of the precise aspects that influence behaviour. The Behaviour Change Techniques Taxonomy v1 (BCTTv1) [16] is a framework for classifying BCTs, which are the ‘active ingredients’ of behaviour change interventions. The taxonomy defines 93 distinct BCTs, grouped into categories. There is no explicit category that relates to social norms. For this review, five BCTs were considered to involve social norms: ‘*6.2. Social Comparison’*, *‘6.3. Information about Others’ Approval’*, *‘9.1. Credible Source’*, *’10.4. Social Reward’*, and *’10.5. Social Incentive’.* The numbers follow the BCTTv1 labelling and definitions are listed in Table 1.

**Table 1:** Definitions of social norms (SN) BCTs and other (non-SN) BCTs that feature prominently in SOCIAL review

SN/non-SN BCT	Name and definition from BCT taxonomy (reproduced from the BCT taxonomy [16])	SOCIAL review name and definition (reproduced from the SOCIAL protocol [17])
Social norm BCT	*6.2. Social comparison* Draw attention to others' performance to allow comparison with the person's own performance. Note: being in a group setting does not necessarily mean that social comparison is actually taking place. Show the doctor the proportion of patients who were prescribed antibiotics for a common cold by other doctors and compare with their own data.	Coded as per original definition, unchanged.
Social norm BCT	*6.3. Information about others’ approval* Provide information about what other people think about the behaviour. The information clarifies whether others will like, approve or disapprove of what the person is doing or will do. Tell the staff at the hospital ward that staff at all other wards approve of washing their hands according to the guidelines.	Coded as per original decision, unchanged.
Social norm BCT	*9.1. Credible source* Present verbal or visual communication from a credible source in favour of or against the behaviour. Note: code this BCT if source generally agreed on as credible, e.g. health professionals, celebrities or words used to indicate expertise or leader in field and if the communication has the aim of persuading. Present a speech given by a high-status professional to emphasise the importance of not exposing patients to Unnecessary radiation by ordering X-rays for back pain.	Coded as per original decision, unchanged.
Social norm BCT	*10.4. Social reward* Arrange verbal or non-verbal reward if and only if there has been effort and/or progress in performing the behaviour (includes ‘positive reinforcement’). Congratulate the person for each day they eat a reduced fat diet.	Changed: Arrange praise, commendation, applause or thanks if and only if there has been effort and/or progress in performing the behaviour (includes ‘positive reinforcement’). New example, relevant to healthcare worker context: arrange for a family doctor to be sent a thank you note for each week that they reduce their level of antibiotic prescribing. Reason for change: the definition of social reward as ‘verbal or non-verbal reward’ is insufficient to distinguish a ‘social’ reward from other types of reward. Further, in the present study, we are interested in only those social rewards that rely on social norms. Praise, commendation, applause or thanks are all injunctive norms messages, providing the target with information about the values, beliefs or attitudes of the reference group, conveying social approval or disapproval.
Social norm BCT	*10.5 Social incentive* Inform that a verbal or non-verbal reward will be delivered if and only if there has been effort and/or progress in performing the behaviour (includes ‘positive reinforcement’). Inform that they will be congratulated for each day that they eat a reduced fat diet.	Changed: Inform that praise, commendation, applause or thanks will be delivered if and only if there has been effort and/or progress in performing the behaviour (includes ‘positive reinforcement’). New example, relevant to healthcare worker context: Promise a family doctor in advance that they will be sent a thank you note for each week that they reduce their level of antibiotic prescribing. Reason for change The definition of social reward as ‘verbal or non-verbal reward’ is insufficient to distinguish a ‘social’ reward from other types of reward. Further, in the present study, we are interested in only those social rewards that rely on social norms. Praise, commendation, applause or thanks are all injunctive norms messages, providing the target with information about the values, beliefs or attitudes of the reference group, conveying social approval or disapproval.
Other BCT (not social norm)	*7.1. Prompts and cues* Introduce or define environmental or social stimulus with the purpose of prompting or cueing the behaviour. The prompt or cue would normally occur at the time or place of performance Note: when a stimulus is linked to a specific action in an if-then plan including one or more of frequency, duration or intensity also code *1.4, Action planning*. Put a sticker on the bathroom mirror to remind people to brush their teeth	Coded as per original definition, unchanged.
Other BCT (not social norm)	*3.1. Social support (unspecified)* Advise on, arrange or provide social support (e.g. from friends, relatives, colleagues, buddies or staff) or non-contingent praise or reward for performance of the behaviour. It includes encouragement and counselling, but only when it is directed at the behaviour. Note: attending a group class and/or mention of ‘follow-up’ does not necessarily apply this BCT, support must be explicitly mentioned; if practical, code *3.2, Social support (practical)*; if emotional, code *3.3, Social support (emotional) (includes ‘Motivational interviewing’ and ‘Cognitive Behavioural Therapy’).* Advise the person to call a ‘buddy’ when they experience an urge to smoke. Arrange for a housemate to encourage continuation with the behaviour change programme. Give information about a self-help group that offers support for the behaviour.	Coded as per original definition, unchanged.
Other BCT (not social norm)	*4.1. Instructions on how to perform the behaviour* Advise or agree on how to perform the behaviour (includes ‘*Skills training’*). Note: when the person attends classes such as exercise or cookery, code *4.1, Instruction on how to perform the behaviour*, *8.1, Behavioural practice/rehearsal* and *6.1, Demonstration of the behaviour.* Advise the person how to put a condom on a model of a penis correctly	Coded as per original definition, unchanged.
Other BCT (not social norm)	*5.1. Information on Health Consequences* Provide information (e.g. written, verbal, visual) about health consequences of performing the behaviour. Note: consequences can be for any target, not just the recipient(s) of the intervention; emphasising importance of consequences is not sufficient; if information about emotional consequences, code *5.6, Information about emotional consequences*; if about social, environmental or unspecified consequences code *5.3, Information about social and environmental consequences*.	Coded as per original definition, unchanged.

The aim was to conduct a systematic review to assess the impact on healthcare workers’ compliance with professional practice recommendations of interventions delivering social norms BCTs, compared to controls. Two research questions were addressed:

1. What is the effect of interventions containing social norms BCTs on (a) the clinical behaviour of healthcare workers, and (b) resulting patient health outcomes?
2. Which contexts, modes of delivery and behaviour change techniques are associated with the effectiveness of social norms interventions on healthcare worker clinical behaviour change?

## Methods

The study design was a systematic review with meta-analysis [18], meta-regression [19] and network meta-analysis [20]. This paper follows the Preferred Reporting Items for Systematic Reviews and Meta-Analyses (PRISMA) statement [21]. Six members of the public attended workshops to discuss the relevance of the review to patients and carers, study design and dissemination. The group felt that patients can potentially have a role in changing healthcare worker behaviour, for example by reminding healthcare workers to wash their hands; or telling the General Practitioner (GP) they do not want antibiotics for a cold, although they were cynical about whether doctors would listen. In response, we changed our data collection to record whether any studies considered patients’ role in social norms interventions. Their advice on how to interpret our results to a broad audience will influence our future dissemination plans. An independent study steering committee, including a member of the public, provided encouragement and counsel throughout the project.

### Protocol and registration

The study was registered on PROSPERO (CRD42016045718) and a protocol is available [17].

### Searches

A search strategy was developed, following an iterative process of scoping searches. In July 2018, searches were undertaken in MEDLINE, PsycINFO, EMBASE, CINAHL, BNI, Cochrane CENTRAL and Web of Science (see Appendix 1). Backward and forward citation searching was not conducted, as per the protocol, due to time constraints.

### Study inclusion criteria

Studies were included if they met the criteria in Table 2.

**Table 2:** Inclusion criteria

PICOS criterion	Description
**Population**	Healthcare workers, including managers and those in training.
**Intervention**	A social norms intervention in a (non-simulated) healthcare setting that seeks to change the clinical behaviour of target population by exposing them to the values, beliefs, attitudes, or behaviours of a reference group or person.
**Comparison/control**	No restrictions on the comparators.
**Outcomes**	Primary outcome of interest was compliance with the desired clinical behaviour. Secondary outcomes were patient health-related outcomes.
**Study design**	Randomised controlled trials published in peer-reviewed journals, in English Language. Grey literature was not eligible for inclusion.

### Screening

Covidence was used to facilitate screening and data extraction [22]. One reviewer screened all titles and abstracts against the inclusion criteria; a second reviewer screened a 20% random sample to assess reliability. Studies included to the full-text stage were independently screened by two researchers. Any disagreements were resolved through discussion, moderation of a third researcher or team review.

### Data extraction

Data from included studies were extracted using a tailored data extraction form (Appendix 2) [23]. Information relating to the population and setting, methods, participant characteristics, intervention characteristics (delivery and BCT content), comparators, outcomes and results were extracted.

For the primary outcome (healthcare worker clinical behaviour), we extracted all available summary data on compliance of the healthcare worker with the desired behaviour at the time point closest to 6 months post-randomisation. Where multiple measures of compliance were reported we followed this list of priorities: (a) reported in sufficient detail to calculate standardised mean difference, (b) observed rather than self-report, (c) appropriate adjustment for clustering, (d) continuous measure, (e) final score rather than change from baseline, (f) described as primary outcome, (g) used to calculate sample size and (h) reported first. A similar approach was followed for patient health outcomes.

All identified BCTs (including both social norms and non-social norms) in all control and intervention arms of included studies were independently coded by two trained researchers using the BCTTv1 [16] and recorded on a BCT extraction form (Appendix 3). The intervention descriptions from all relevant papers (including protocols, process evaluations or additional sources cited in the included studies) were coded to capture the BCTs as closely as possible. Inter-rater reliability for each of the BCTs that were present at least once across all arms was assessed using the prevalence and bias-adjusted kappa (PABAK) statistic (see Appendix 4), which adjusts for both the prevalence and occurrence of BCTs [24]. In circumstances where prevalence is low, the widely used chance-corrected kappa statistic is likely to underestimate reliability as it is highly dependent on prevalence [25].

### Study quality assessment

Risk of bias was independently assessed by two researchers using the Cochrane Collaboration risk of bias tool. The percentages of high/low/unclear judgements for each criterion across included studies were calculated.

### Data analysis/synthesis

Any observed measure of healthcare worker behaviour was converted into a standardised mean difference (SMD, Cohen’s *D*) comparing intervention and control groups [26]. Odds ratios were converted to SMDs [27]. Where necessary, the sign of the SMD was changed to ensure that a positive SMD represented an improvement in compliance with the desired behaviour.

Where data were from appropriately analysed cluster randomised trials or stepped wedge trials the reported adjusted standard errors were used. Where adjusted standard errors were not reported, we inflated them ourselves to account for clustering [28].

Where data were missing, we searched for companion papers. Missing standard deviations were estimated using any available information (e.g. *p* values, confidence intervals, range, interquartile range) or by searching for trials with similar outcome measures. For cluster randomised trials, we estimated the intracluster correlation coefficient (ICC) where necessary by taking the average of results from similar studies.

Where studies, including factorial trials, assessed more than one intervention, data were extracted for any comparisons that were relevant to the review, avoiding double-counting by dividing the number of participants in the control arm evenly between comparisons. Where there was more than one control arm, the comparison that was the purest test of a social norms intervention was utilised. Where a study was an appropriately analysed factorial trial the covariate and standard error that best estimated the effect of a social norms intervention was extracted.

All studies that reported a primary or secondary outcome measure that could be converted into an SMD were included in meta-analyses. The approach to utilising the five social norms BCTs in the analysis was to subtract the control arm BCTs from those in the intervention arm, to identify those BCTs that were the active ingredients being tested in the trial. The BCT *feedback on behaviour* was present alongside a social norm BCT in 88 of 100 comparisons and so we combined *feedback on behaviour* with the social norm BCT with which it appeared for the purpose of primary meta-analyses.

Fixed effects meta-analysis [29] and forest plots, stratified by BCT were used to assess the effect of social norms on the clinical behaviour of healthcare workers and patient health outcomes. Sources of variation in the type of social norm, context and mode of delivery were explored using both exploratory subgroup analysis and meta-regression [30]. Network meta-analysis [20] was used to (a) utilise all available data and therefore maximise power by including trials that compared two or more different types of social norms (in addition to those that compared a social norm intervention to a control) and (b) rank the different types of social norms intervention in order of effectiveness. A fixed effects approach to meta-analysis was adopted to yield a summary of the evidence in these trials (i.e. the average effect), rather than an estimate of a common underlying treatment effect. Random effects analyses are also reported.

Pre-planned sensitivity analyses assessed the robustness of the conclusions by excluding studies: at high risk of bias on key domains (allocation concealment, sequence generation, selective outcome reporting, attrition, other biases); with ‘mean percentage’ < 20% or > 80% (due to expected skewed distribution)’ with imputed standard deviations; using estimated ICCs; with and without feedback on desired behaviour.

## Results

### Study characteristics

**Fig. 1:**
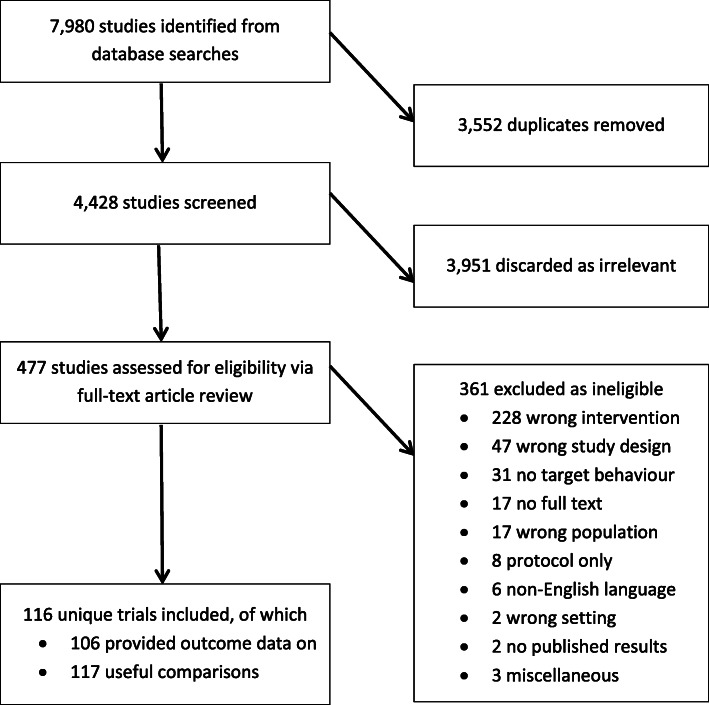
PRISMA diagram

There were 4428 citations screened at the title and abstract stage; 477 full-text papers were screened, of which 116 unique trials met the inclusion criteria. Ten of these trials did not report usable outcome data; therefore, a total of 106 trials contributed findings to the review (Fig. 1, Appendix 5). Some studies had more than one trial arm, resulting in 117 included comparisons. The trial and intervention characteristics are summarised in Table 3, and characteristics of each individual comparison are provided in Appendix 6. There were 100 comparisons suitable for meta-analysis. These included studies testing *social comparison* (*n* = 79) *credible source* (*n* = 7) and *social reward* (*n* = 2) against control. Other studies tested more than one social norm together: *social comparison* and *credible source* (*n* = 6), *social comparison* and *social reward* (*n* = 2), multiple social norms (more than two) together (*n* = 4). Over half of the included trials were conducted in North America; most studies were set in primary care and hospitals, targeting doctors. A broad range of behaviours were targeted including prescribing, management of conditions and test ordering. Two thirds of the trials were cluster RCTs. The interventions were delivered in a variety of formats; a third was delivered on one occasion and the rest on multiple occasions. Most were delivered by someone outside of the target organisation, often an investigator, and three quarters aimed to increase, rather than decrease the behaviour. Some intervention characteristics were poorly reported; format and frequency of delivery were missing in a third of studies (Table 3).

**Table 3:** Characteristics of included studies

Study characteristic (***n*** = 106)	No.	%	Study characteristic (***n*** = 106)	No.	%	Intervention characteristic (***n*** = 117)	No.	%
**Country**			**Type of trial**			**Source**		
Australia	8	7.5	Cluster RCT	69	65.1	Peer	6	5.1
Canada	15	14.2	Factorial	4	3.8	Investigators	83	70.9
Denmark	4	3.8	Randomised controlled trial	28	26.4	Supervisor or senior colleague	2	1.7
UK	13	12.3	Stepped wedge	4	3.8	Patient	1	0.9
Netherlands	6	5.7	Matched pairs, cluster RCT	1	0.9	*Credible source*	15	12.8
USA	45	42.5	**Low baseline performance?**^**a**^			Other	1	0.9
Other/multiple	15	14.2	No	103	97.2	Not reported	9	7.7
**Setting**			Yes	2	1.9	**Internal/external delivery** ^**b**^		
Primary (GP/GP practice nurses)	57	53.8	Unclear	1	0.9	Internal	17	14.5
Hospital (inpatient and outpatient)	31	29.3				External	81	69.2
Community	4	3.8				Unclear/not reported	19	16.2
Care/nursing home	4	3.8				**Reference group**		
Mixed	7	6.6				Peer	97	82.9
Other	3	2.8	**Intervention characteristic (*n*** **= 117)**	**No.**	**%**	Professional body	1	0.9
**Type of HCP**			**Format**			Senior person	9	7.7
Doctor (primary care)	45	42.5	Face-to-face meeting	16	13.7	Patient(s)	1	0.9
Doctor (secondary)	19	17.9	Email	10	8.5	Multiple	4	3.4
Other (nurse/dentist/AHP/pharmacist)	7	6.6	Written (paper)	29	24.8	Unclear/not reported	5	4.3
Mixture/whole team	35	33.0	Separate computerised	10	8.5	**Direction of change**		
**Target behaviour**			Mixed	18	15.4	Increase	85	72.6
Prescribing (incl. vaccinations)	40	37.7	Unclear/not reported	34	29.1	Decrease	30	25.6
Handwashing/hygiene	4	3.8	**Frequency**			Maintenance	0	0.0
Tests/assessments	21	19.8	Only once	35	29.9	Unclear	2	1.7
Referrals	3	2.8	Twice	10	8.5	**Comparator**		
Management communications	25	23.6	More than twice	45	38.5	Alternative intervention	15	12.8
Other	2	1.9	Unclear/not reported	27	23.1	Usual practice	59	50.4
Multiple	11	10.4				Attention or waitlist control	18	15.4
						Concomitant intervention^c^	25	21.4

^a^Does the inclusion criteria target participants based on low target performance?

^b^The person delivering the intervention internal or external to the target person’s organisation?

^c^Intervention that appears in both arms

### Effects of interventions

#### Overall effects on clinical behaviours and patient outcomes

Combined data from fixed effects meta-analysis suggested that social norms interventions were associated with an improvement in healthcare worker clinical behaviour of 0.08 SMDs (95%CI 0.07 to 0.10, *n* = 100 comparisons), and an improvement in patient health outcomes of 0.17 SMD (95%CI 0.14 to 0.20), on average. There was a large amount of heterogeneity with an overall *I*^2^ value of 85.4% (primary) and 91.5% (secondary) suggesting that some studies reported substantially higher or lower effects than the average. However, *I*^2^ is related to precision and rapidly approaches 100% when the number of studies is high [31]. Similar conclusions were drawn from random effects meta-analysis an overall improvement in healthcare worker clinical behaviour of 0.16 SMD (95%CI 0.11 to 0.21, *I*^2^ = 85.4%, τ^2^ = 0.043). Note that the random effects analysis was associated with a larger effect size and wider confidence interval because more weight is given to smaller trials. These results remained robust after all of our pre-planned sensitivity analyses (Appendix 7).

#### Social norms behaviour change techniques

**Fig. 2:**
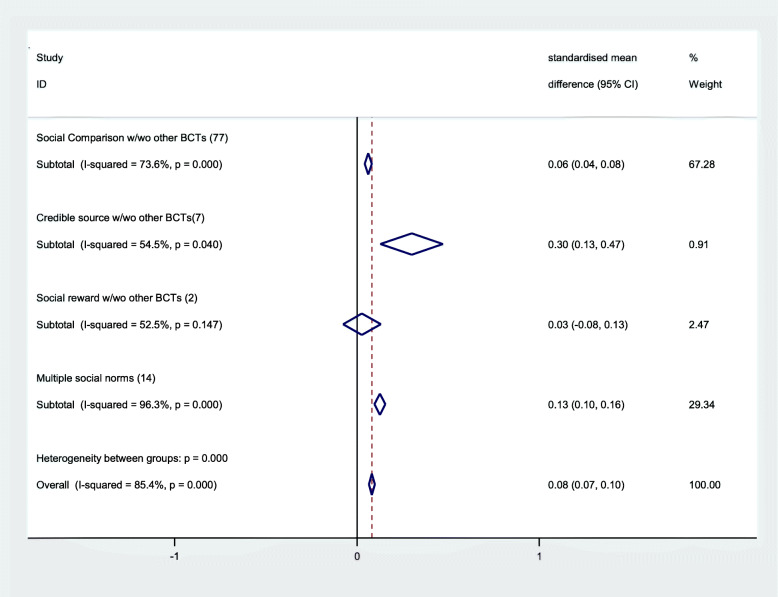
Fixed effects forest plot summarised by alternative categorisation of BCTs

Meta-analysis, stratified by social norms BCTs indicated that two of the social norms BCTs had a positive effect on healthcare worker clinical behaviour (Fig. 2): *credible source* (with or without other BCTs) (SMD 0.30, 95%CI 0.13 to 0.47, *n* = 7) and *social comparison* (with or without other BCTs) (SMD 0.06, 95%CI 0.04 to 0.08, *n* = 77). *Social reward* may not be effective (SMD 0.03, 95%CI − 0.08 to 0.13, *n* = 2), based on a small sample. We did not find sufficient evidence to examine the effect of the other two social norm BCTs (*information about others’ approval* and *social incentive*). Multiple social norms delivered together were also effective on average (SMD 0.13, 95%CI 0.10 to 0.16). When we looked at the most common combinations of social norms BCTs alongside other BCTs, three types of social norms intervention were most effective, on average, compared to control (Table 4): *credible source* (0.30, 95%CI 0.13 to 0.47); *social comparison* combined with *social reward* (0.39, 95%CI 0.15 to 0.64); and *social comparison* combined with *prompts and cues* (0.33, 95%CI 0.22 to 0.44). *Social comparison* delivered with *credible source* (0.16, 95%CI 0.12 to 0.19), on its own (0.05, 95%CI 0.03 to 0.08) or with *social support (unspecified)* (SMD 0.10, 95%CI 0.04 to 0.16) were all effective, on average, compared to control. This was confirmed by network meta-analysis. Table 5 shows the different contexts and settings for the social norms BCTs and there does not appear to be any obvious patterns of use of the BCTs in particular contexts: social comparison, credible source and social reward are each used in multiple different contexts either alone or alongside other BCTs. Regression analysis suggests that results were consistent even after adjustment for context and setting. Illustrative case studies providing examples of the three intervention types found to be most effective (credible source, social comparison with prompts/cues, social comparison and social reward) are shown in Table 6.

**Table 4:** Intervention effects calculated from meta-analysis and network meta-analysis, ordered by effect size (intervention v control)

Type of social norms intervention	Number of comparisons for meta-analysis (network meta-analysis)	SMD meta-analysis (95%CI) ***n*** = 100	SMD network meta-analysis (95%CI) ***n*** = 102	Probability of being the best intervention (%)
Social comparison + social reward	2	0.39(0.15 to 0.64)	0.39 (0.15 to 0.64)	59.2
Social comparison + prompts/cues	5	0.33(0.22 to 0.24)	0.33 (0.22 to 0.44)	22.2
Credible source^a^	7	0.30(0.13 to 0.47)	0.30 (0.13 to 0.47)	18.6
Social comparison + credible source^a^	8(10)	0.16(0.12 to 0.19)	0.16(0.12 to 0.20)	0.0
Social comparison + social support (unspecified)	7	0.10(0.04 to 0.16)	0.10 (0.04 to 0.16)	0.0
Other multiple social norms BCTs	4	0.07(0.03 to 0.12)	0.07(0.03 to 0.12)	0.0
Social comparison	33(35)	0.05(0.03 to 0.08)	0.05(0.03 to 0.08)	0.0
Social comparison + other BCTs	23	0.04(0.00 to 0.08)	0.04(0.00 to 0.08)	0.0
Social reward	2	0.03(− 0.08 to 0.13)	0.03(− 0.08 to 0.13)	0.0
Social comparison + instructions on how to perform the behaviour + prompts/cues	5	0.01(− 0.10 to 0.11)	0.01(− 0.10 to 0.11)	0.0
Social comparison + info on health consequences	4	− 0.14(− 0.33 to 0.05)	− 0.14(− 0.33 to 0.05)	0.0

^a^With/without other BCTs

**Table 5:** Key trial characteristics by type of comparisons

Type of comparison	Test of SC	Test of CS	Test of SR	Test of SC + CS	Test of SC + SR	Test of SC + social support (unspecified)	Test of SC + prompts and cues	Test of SC + info on health consequences	Test of SC + instructions + prompts/cues	Test of SC + others BCTs	Test of CS + other BCTs	Test of SC + CS + other BCTs	Test of SR +other BCTs	Test of multiple SNs + other BCTs
**Number of comparisons with primary outcome data**	33	3	1	2	2	7	5	4	5	25	4	4	1	4
**Target Behaviour**														
Prescribing	15(45%)		1(100%)	1(50%)	2(100%)	2(29%)	1(20%)	3(75%)	1(20%)	11(44%)	1(25%)	1(25%)		1(25%)
Hand/hygiene												1(25%)	1(100%)	1(25%)
Tests	7(21%)					1(14%)	3(60%)	1(25%)	3(60%)	4(16%)	1(25%)	1(25%)		
Referrals										2(8%)		1(25%)		
Man/comm	5(15%)	3(100%)		1(50%)		2(29%)			1(20%)	5(20%)	2(50%)			2(50%)
Other						12(14%)				1(4%)				
Multiple	6(18%)					1(14%)	1(20%)			2(8%)				
**Type of HCP**														
Doctor GP	16(48%)			1(50%)	2(100%)	4(57%)	2(40%)	1(25%)	4(80%)	11(44%)	2(50%)	1(25%)		1(25%)
Doctor secondary	4(12%)	3(100%)				1(14%)	1(20%)	1(20%)		2(12%)	1(25%)	1(25%)		1(25%)
Other HCP	4(12%)		1(100%)							1(4%)	0(0%)			1(25%)
Mixed/team	9(27%)			1(50%)		2(29%)	2(40%)	2(40%)	1(20%)	10(40%)	2(50%)	2(50%)	1(100%)	1(25%)
**Setting**														
Primary	18(55%)			1(50%)	2(100%)	4(57%)	4(80%)	1(25%)	5(100%)	17(68%)	2(50%)	1(25%)		1(25%)
Hospital	6(18%)	3(100%)				2(29%)	1(20%)	2(50%)		6(24%)	2(50%)	2(50%)	1(100%)	2(50%)
Community	1(3%)		1(100%)	1(50%)										1(25%)
Care/nursing	0(0%)					1(14%)		1(25%)		1(4)		1(25%)		
Mixed	7(21%)													
Other	1(3%)													

*SC* social comparison, *CS* credible source, *SR* social reward

**Table 6:** Case studies—summary descriptions of interventions for example studies of the three intervention types found to be most effective

Study *Trial design* *Target healthcare worker*	Aims	Outcome measure SMD(95% CI)	Control arm	Intervention description
***Credible source + social comparison***				
Hallsworth et al. (2016) [32] *RCT* *Doctor (primary care)*	To reduce the number of unnecessary prescriptions of antibiotics by GPS in England	The rate of antibiotic items dispensed per 1000 population 0.13 (0.03 to 0.29)	Delayed intervention (after the end of the trial *(no BCTs were coded).*	A letter was sent to GPs from the Chief Medical Officer. The letter stated that the practice was prescribing antibiotics at a higher rate than 80% of practices in its NHS Local Area Team, and used three concepts from the behavioural sciences. The first was social norm information about how the recipient’s practices prescribing rate compared with other practices in the local area. Second, the letter was addressed from a high-profile figure with the assumption that this would increase the credibility of its content. Finally, the letter presented three specific, feasible actions that the recipient could do to reduce unnecessary prescriptions of antibiotics: giving patients advice on self-care, offering a delayed prescription and talking about the issue with other prescribers in his or her practice. The letter was accompanied by a copy of the patient-focused “Treating your infection” leaflet, which acted to reinforce the message of the letter by supporting delayed or reduced prescribing. *(9.1 Credible source, 6.2 Social comparison, 2.2 Feedback on behaviour, 4.1 Instruction on how to perform the behaviour).*
***Social comparison + prompts/cues***				
Vellinga et al (2016) [33] Arm A *Cluster RCT* *Doctor-GP*	To increase the number of first-line antimicrobial prescriptions for suspected urinary tract infections (UTIs) in adult patients	Adherence to guidelines for antimicrobial prescribing in primary care 0.55 (0.32 to 0.77)	Phase 1—a coding workshop: routine coding for UTIs using standardised codes were demonstrated. The purpose of this was to facilitate the generation of electronic audit and feedback reports (not available to control until after the trial). Control practices then provided 'usual care’ for the remainder of the intervention (*no BCTs were coded).*	Arm A: phase 1—a coding workshop (same as control). Phase 2—interactive workshops were designed to promote changes in antimicrobial prescribing for the treatment of UTIs by presenting an overview of prescribing and antimicrobial resistance, discussing the role of the GP in the spread of anti-microbial resistance. A computer prompt was developed for use within the selected GP practice management software system. This prompt summarised the recommendations for first-line antimicrobial treatment and appeared on the computer screen when the GP entered the International Classification of Primary Care code (U71) for 'cystitis, urinary infection, other’. This prompt also reminded the GP to collect patients’ mobile telephone numbers. Electronic audit and feedback reports were available to download by GPs. These reports provided the practice with information on antimicrobial prescribing for UTI in comparison with the aggregated information from the other practices participating in the intervention. *(7.1 Prompts/cues, 2.2 Feedback on behaviour, 6.2 Social comparison)*
***Social comparison + social reward***				
Persell et al. (2016) [34] *2 × 2 × 2 Factorial* *Doctor (GP)*	To reduce inappropriate antibiotic prescribing for acute respiratory infections (ARIs)	Physician rate of oral antibiotic prescribing for non-antibiotic-appropriate ARIs, acute sinusitis/pharyngitis and all other diagnoses of respiratory infection SMD 0.44 (− 0.06 to 0.94)	Intervention 1 (accountable justifications): Clinicians received electronic health record (EHR) alerts summarising the treatment guidelines corresponding to the ARI diagnosis for which the antibiotic was being written, prompted the clinician to enter a free-text justification for prescribing an antibiotic, and informed the clinician that the free-text justification provided would be included in the patient’s medical record where it would be visible to other clinicians. Clinicians were also informed that if no free-text justification was entered, a default statement “No justification for prescribing antibiotics was given” would appear in the record. If the antibiotic order was cancelled, no justification was required, and no default text appeared. Alerts were suppressed for patients with comorbid chronic conditions that exempted these patients from clinical guidelines *(4.1 Instruction on how to perform the behaviour*, *7.1 Prompts/cues)* Intervention 2 (suggested alternatives): when entering an ARI diagnosis for a patient, clinicians received a computerized alert containing multiple non-antibiotic prescription and non-prescription medication choices as well as educational materials that could be printed and given to the patient. *(7.1 Prompts/cues)*	Intervention 3 (peer comparison): clinicians received emailed monthly performance feedback reports which included the clinician’s individual antibiotic prescribing rates for non-antibiotic-appropriate ARIs and as a benchmark, the antibiotic prescribing rate for clinicians who were at the 10th percentile within the clinic (i.e. the lowest rates of inappropriate antibiotic prescribing). If clinicians were among the 10% of their peers with the lowest prescribing rates the emailed reports told clinicians "You are a top performer.” If clinicians were not among the 10% best, the emailed report told clinicians “You are not a top performer. You are prescribing too many unnecessary antibiotics”. The proportion of “top performers” could be greater than 10 % of clinicians if more than 10 % of clinicians had an inappropriate antibiotic prescribing rate of 0*. (2.2 Feedback on behaviour, 6.2 Social comparison, 10.4 Social reward)*

#### Context and mode of delivery

Meta-analysis suggested that social norms interventions were effective in a variety of different contexts. The effect was seen with doctors on average (SMD 0.08, 95%CI 0.07 to 0.10, *n* = 68) and other healthcare workers (SMD 0.08, 95%CI 0.04 to 0.12, *n* = 12), but not with nurses and allied healthcare workers (SMD − 0.01, 95%CI − .012 to 0.11, *n* = 5). They appeared successful across a range of clinical behaviours, including prescribing (SMD 0.11, 95%CI 0.09 to 0.13, *n* = 21), arranging, conducting or administering tests/assessments (SMD 0.10, 95%CI 0.06 to 0.13, *n* = 21), and management and communication around health conditions (SMD 0.06, 95%CI 0.01 to 0.12, *n* = 23), but may be less effective with handwashing (SMD 0.04, 95%CI − 0.05 to 0.13, *n* = 3) and referrals to other health services (SMD − 0.08, 95%CI − 0.23 to 0.07, *n* = 3). The effects were similar in primary (SMD 0.07, 95%CI 0.05 to 0.09, *n* = 56) and secondary care (SMD 0.12, 95%CI 0.07 to 0.18, *n* = 27) but may be less effective in community (SMD 0.02, 95%CI − 0.05 to 0.10, *n* = 4) and care home (SMD 0.03, 95%CI − 0.05 to 0.10, *n* = 4) settings. The effect appears to be consistent, regardless of whether a peer benchmark (0.06, 95%CI 0.02 to .011, *n* = 13) or the average (0.11, 95%CI 0.09 to 0.13, *n* = 67) is included. On average, they were slightly less effective in increasing behaviours (e.g. increasing diabetes testing) than at reducing behaviours (e.g. reducing antibiotic prescriptions). The effect was similar regardless of who delivered the intervention and whether it came from within the organisation or from an external source. Interventions that were delivered once (0.25, 95%CI 0.21 to 0.30, *n* = 28) were more effective than those delivered more frequently (0.06, 95%CI 0.04 to 0.08, *n* = 47). Delivery by website was most effective (0.23, 95%CI 0.15 to 0.31, *n* = 8); delivery by email, in writing, and in mixed format were all consistent with the average effect, but face-to-face appeared to be ineffective (− 0.01, 95%CI − 0.06 to 0.03, *n* = 14). The number of studies in some of these categories was low (nurses and allied healthcare workers, handwashing, referrals to other services, community and care homes), and none of the pre-planned covariates for context and setting appeared to explain much of the heterogeneity in meta-regression, suggesting that any conclusions about context and mode of delivery should remain cautious.

#### Risk of bias

**Fig. 3:**
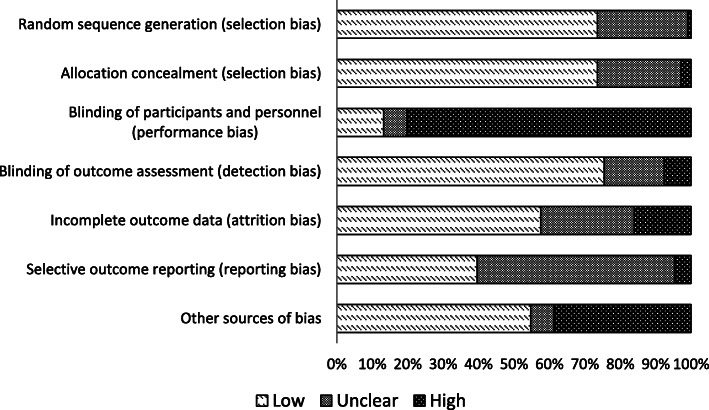
Review authors’ judgements about each risk of bias item (%)

**Fig. 4:**
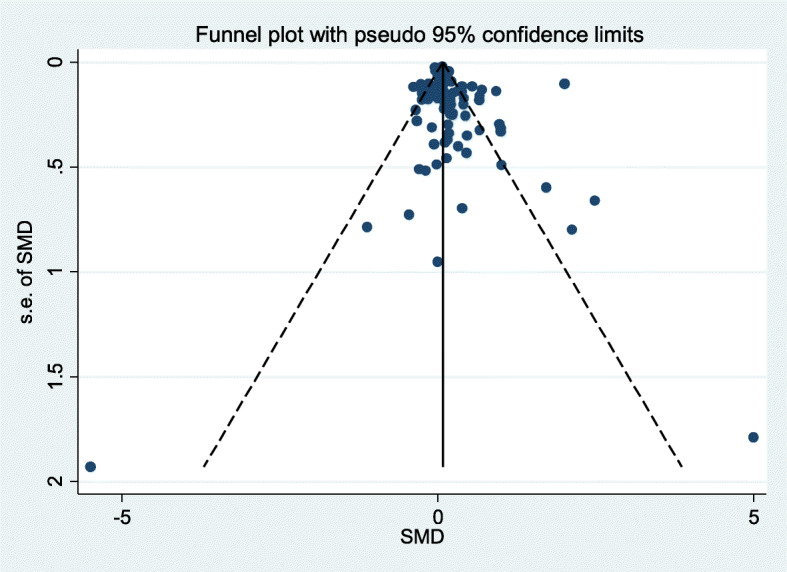
Funnel plot

A summary of each risk of bias item across the studies is shown in Fig. 3. Risk of bias was high in 80% of trials for the blinding of participants and personnel domain and so we cannot rule out the possibility of response bias. This high risk of bias was mainly due to the nature of the interventions (i.e. many of the studies were cluster trials, randomised at the hospital or clinic level, making blinding impractical). In a sensitivity analysis restricting the meta-analysis to trials at low risk of bias for each key domain, the overall treatment effect changed little, suggesting the results were robust. There were five studies at high risk of bias for outcome reporting and 59 with unclear risk of bias. A funnel plot (Fig. 4) identified that the review may be missing some unpublished negative trials, or including more positive trials than expected, suggesting selective outcome reporting.

## Discussion

### Summary of evidence

Social norms interventions can be an effective approach to changing the clinical behaviours of healthcare workers. Meta-analysis showed social norms interventions were associated with an improvement in healthcare worker clinical behaviour outcomes of 0.08 SMDs (95%CI 0.07 to 0.010, *n* = 100 comparison) and an improvement in patient health outcomes of 0.17 SMD (95%CI 0.14 to 0.20, *n* = 14 comparisons), on average.

There was a large amount of heterogeneity, with some studies reporting substantially higher or lower effects. There was strong evidence from multiple studies that interventions involving *social comparison* or *credible source,* with and without other BCTs, were effective on average, both separately and together. *Social comparison* is effective when combined with various other BCTs including *social support (unspecified)* but it appears to be most effective when combined with *prompts/cues*. *Social reward* appeared not to be effective when used alone but had an above-average effect when combined with *social comparison.* The effect of social norms interventions remained clear in the meta-regression, even after taking into account context and setting.

Meta-analyses exploring context and delivery showed that social norms were effective with a variety of healthcare workers, in primary and secondary care, and across a range of clinical behaviours. On average, social norms interventions were more effective for reducing than increasing behaviours. Interventions appeared equally effective regardless of whether they came from an internal or external source. In contrast to previous studies [14], delivering the intervention once appeared to be more effective than frequent delivery: one explanation for this, which warrants further investigation, is whether frequent delivery is associated with attempts to change intractable behaviours.

Sensitivity analyses found the overall treatment effect to be robust and not strongly influenced by trials which scored high/unclear risk of bias across key domains. There is a possibility of response bias due to lack of blinding. While it is difficult to blind healthcare workers in these trials, there were examples where the risk of response bias was minimised, e.g. cluster trials where the healthcare worker was not informed of the existence of the trial.

### Discussion of findings in relation to the literature

A Cochrane systematic review (*n* = 140) of the effect of A&F on healthcare worker behaviour and patient health outcomes [14] found a wide variation in the effect of A&F and recommended future research to explore how this variation, related to the intervention design, context and recipient [11]. The results of our review contribute to this agenda by suggesting aspects of the design of A&F interventions that are associated with positive outcomes: (1) highlighting that a *credible source* approves of the desired behaviour; (2) feedback on an individual’s behaviour is likely to be more effective if accompanied by *social comparison*; (3) complex interventions involving multiple social norms seem to be effective; (4) *social comparison* seems to be enhanced by the use of *prompts and cues*, such as computerized pop-ups recommending actions to GPs when particular symptoms or diagnoses are entered into an electronic system [35], but the benefit of *prompts and cues* may only hold when the healthcare worker understands how to do the behaviour. The effects of social norms were reasonably consistent across a range of healthcare workers, behaviours and settings. In contrast to an earlier review of A&F [14], delivering the intervention once appeared to be sufficient and sending the intervention by website or other computerised format was most effective. Our results align with findings from a recent synthesis of qualitative literature on A&F which found that letting healthcare workers know how their performance relates to that of their peers (*social comparison*) and providing opportunities for peer discussion (*social support (unspecified)*) were valuable in changing behaviour [6]. However, our finding that face-to-face interventions were less effective than remotely delivered interventions contrasts with results for meta-analyses of smoking cessation interventions where personalised interventions were associated with greater effectiveness [36]. Recent literature suggests that de-implementation is often even more challenging than implementation due to a number of psychological biases: health professionals tend to focus on information that confirms their established beliefs; people are more concerned about losses than gains; and a sense of professional autonomy strengthens attachment to established practices [37, 38]. Given the challenges of de-implementation our finding that social norms interventions were more effective in increasing behaviour than decreasing it are perhaps not surprising.

A recent overview of 67 systematic reviews on promoting professional behaviour change in healthcare found that the most effective interventions were educational outreach using academic detailing, A&F and reminders [39]. Using normalization process theory as a theoretical lens, the authors concluded that interventions that seek to ‘restructure and reinforce new practice norms’ (opinion leaders, educational meetings and materials/guidelines) and those which ‘associate practice norms with peer and reference group behaviours’ (including A&F and academic detailing, where a target healthcare worker receives individual support or advice from someone else with expertise in that area) are most likely to be successful in changing clinical behaviour. Combining the two approaches together may be particularly effective, by creating clear rules of conduct and encouraging individuals to follow their peers [39]. Interventions that seek to change attitudes were less likely to be successful. The importance given to peer and reference group behaviours in this previous study justifies our efforts to identify which social norms interventions are associated with success.

The effect sizes seen in this review appear to be similar to other reviews of interventions to change health professional behaviour [40]. Baskerville et al found that practice facilitation was associated with an improvement of 0.56 SMD (95% CI 0.43 to 0.68) in guideline adoption in primary care. Baker [41] reported that tailored interventions to overcome barriers to change are associated with an odds ratio for the improvement in professional behaviour of 1.51 (95% CI 1.16 to 2.01) which corresponds to an SMD of approximately 0.24 (95% CI 0.09 to 0.39). The modest effects size seen for social comparison appears in line with that observed by Ivers who found that Audit and feedback improved binary behavioural outcomes by a median of 4.3 percentage points and continuous outcomes by a median of 1.3 percentage points. In a meta-synthesis of systematic reviews of health behaviour change in general, Johnson found effect sizes between 0.08 to 0.45 [42].

### Strengths and limitations

Our search strategy was developed through an iterative process, with input from an Information Scientist. However, it is possible that the strategy may have missed some relevant interventions if social norms BCTs or behaviour change theories were not mentioned in the title or abstract.

We included studies regardless of outcome measure, and we converted any available outcome into a standardized mean difference: this meant we were able to summarise all the available evidence in one analysis. The included trials incorporated a variety of contexts and settings; trial designs and units of analysis. This has led to a heterogeneous review; and we acknowledge the limitations of this approach. The magnitude of effects for the most promising behaviour change interventions were around 0.3 SMDs, which relative to the between study variability τ(0.2) does seem to indicate an important effect.

Trials were excluded from the review where the intervention did not target a specific behaviour: for example, if the intervention was aimed at a healthcare worker with the intention of reducing patient blood pressure, but did not make explicit what behaviour(s) were expected of the healthcare worker to achieve the reduction. These exclusions occurred because, if a behaviour is not specified, it is not possible to determine whether or not an intervention actually targeted that behaviour and change in that behaviour (our primary outcome) cannot be assessed. This approach is consistent with the coding instructions of the BCTTv1 [16]. There is a potential risk that we have excluded some studies where there was a target behaviour but it was poorly reported.

We used the BCTTv1 [16], which has been based on a significant body of research, to code for BCTs that could be associated with the effectiveness of interventions. However, BCTs were only coded based on published reports and we did not ask study authors for intervention manuals due to time constraints. Therefore, it is possible that our coding did not represent all actual BCTs as designed and delivered. The authors of the BCTTv1 have also acknowledged that extension or modification of the BCTTv1 could be appropriate in the future. It is therefore possible that some BCTs that do not yet feature in the BCTTV1 could have been presented alongside social norms BCTs and were missed during the BCT coding exercise.

Ten small studies without suitable outcome measures were omitted from the meta-analysis and some missing information (such as ICCs and standard deviations) were imputed, but sensitivity analyses suggested no significant impact on the review.

The primary approach to meta-analysis was fixed effects [43], which summarises the evidence in these trials, rather than estimating a common underlying treatment effect [44]. This topic is highly contested, so random effects was also undertaken for the most important analyses, as planned. In all analyses the fixed and random effects approaches produced a result in the same direction, with the random effects approach resulting in a higher effect for the intervention because it gives greater weight to smaller studies. The conclusions of the review would be similar, regardless of whether fixed or random effects were used.

All of the meta-analysis was undertaken on the basis of comparisons: the BCTs in the control arm were subtracted from those in the intervention arm to capture BCTs that were actively tested in each study. The active ingredient was what is left of the intervention when the control arm is taken away. This is a suitable approach to examining the effect of the various social norm BCTs, but a limitation is that some interaction effects may have been missed.

The asymmetry of the funnel plot suggested that the review may have missed some unpublished negative trials or be at risk of bias from selective outcome reporting. The resources were not available for translation or to request unpublished material from authors of included studies, so some relevant studies may have been omitted. A single behaviour outcome was selected from every trial using published reports which may have put the review at risk from selective outcome reporting; priority was given to the pre-specified primary outcome. Sensitivity analysis including only those trials with either a relevant pre-specified primary outcome or single relevant behavioural outcome suggested that results were robust to selective outcome reporting.

### Further research

*Credible source* has been identified as an effective intervention component. Yet, it is not commonly used in the health setting to change the behaviour of healthcare workers (only 18% of the comparisons identified in the present review). This may be due to *credible source* lacking formal conceptualisation in the health setting so, whilst it may be used in practice, it is not well-reported. Additional work is needed to develop *credible source* interventions for use in the NHS, such as, whom the target audience would consider as *credible sources*: for example, seniority may not necessarily be perceived as the same as credible. As a first step, a narrative synthesis of the trials using *credible source* in this review, together with the qualitative papers, process evaluations and protocols associated with those trials, would provide further insights into the *credible source* interventions that are associated with more successful outcomes. Qualitative work with healthcare workers, managers and policymakers is also needed to understand the acceptability and feasibility of *credible source*, *social comparison* and *social reward* interventions and to understand who the most credible sources are.

*Social comparison* is currently used more frequently with healthcare workers than *credible source*. We identified a high level of heterogeneity in the effectiveness of *social comparison*. We have started to unravel this heterogeneity, and this research suggests that *social comparison* can successfully be enhanced by the addition of *social reward*, *prompts and cues* or *social support (unspecified);* but further research is warranted. The heterogeneity could potentially be explained by differences in how social comparisons are facilitated and what kind of comparisons are made, and not simply by the combination of BCTs it is delivered with or without. For example, social comparisons may have a different effect depending on the reference frame (e.g. whether one identifies with those compared to) or depending on the direction of the comparison (i.e. upward or downward comparison). Further investigation into the factors that moderate the effect of social comparison is warranted.

The methodological quality of trials was mixed. The review included some large factorial trials that tested several behaviour change interventions simultaneously, which can be an efficient design for exploring different components of behaviour change interventions and their interactions. Multiphase Optimization Strategy may be a useful framework that can be applied to factorial designs for identifying which combination and sequence of components (e.g. BCTs and mode of delivery) can produce optimal outcomes [45]. Some trials also used novel methods to minimize bias such as ‘attention’ controls where participants were given the identical behaviour change intervention for an alternative target behaviour: this type of design is to be encouraged.

## Conclusions

Social norms interventions are an effective method of changing healthcare worker clinical behaviour. Although the overall result is modest and very variable, there is the potential for social norms interventions to be applied at scale and have a significant effect on clinical behaviour and resulting patient health outcomes. Both *credible source* and *social comparison* were effective. *Social comparison* was particularly effective when combined with *prompts and cues*. These interventions were found to be effective in a variety of NHS contexts and across a range of modes of delivery.

## Supplementary Information

**Additional file 1:** **Appendix 1.** Search Strategy. **Appendix 2.** Data Extraction Form. **Appendix 3.** Behaviour Change Techniques (BCTs) Extraction Form. **Appendix 4.** Inter-Rater Agreement for BCT Coding. **Appendix 5.** Included Study References. **Appendix 6.** Study and Intervention Characteristics of Included Comparisons. **Appendix 7.** Sensitivity Analyses.

## Data Availability

The datasets used and analysed during the current study are available from the corresponding author on reasonable request.

## Abbreviations

A&F: Audit and Feedback
BCT: Behaviour Change Technique
BCTTv1: Behaviour Change Techniques Taxonomy Version 1
BNI: British Nursing Index
CI: Confidence Interval
CINAHL: Cumulative Index of Nursing and Allied Health Literature
Cochrane CENTRAL: Cochrane Central Register of Controlled Trials
EMBASE: Excerpta Medica dataBASE
GP: General practitioner
ICC: Intra-class correlation coefficient
MEDLINE: Medical Literature Analysis and Retrieval System Online
MeSH: Medical Subject Headings
PABAK: Prevalence-Adjusted and Bias-Adjusted Kappa
PRISMA: Preferred Reporting items for Systematic Reviews
PROSPERO: The International Prospective Register of Systematic Reviews
RCT: Randomised controlled trial
SMD: Standard mean difference
SN: Social norm
