# Supplementary material for: How effective are social norms interventions in changing the clinical behaviours of healthcare workers? A systematic review and meta-analysis
Source: Implement Sci. 2021 Jan 7;16:8. doi: 10.1186/s13012-020-01072-1 (PMC7792225; doi:10.1186/s13012-020-01072-1)
Supplement: Supplementary file 1 — Additional file 1: Appendix 1. Search Strategy. Appendix 2. Data Extraction Form. Appendix 3. Behaviour Change Techniques (BCTs) Extraction Form. Appendix 4. Inter-Rater Agreement for BCT Coding. Appendix 5. Included Study References. Appendix 6. Study and Intervention Characteristics of Included Comparisons. Appendix 7. Sensitivity Analyses. [file 13012_2020_1072_MOESM1_ESM.docx]

**Appendices**

**Appendix 1 – Search Strategy**

**Ovid MEDLINE search, covered the period 1946 to July 2018, run on 23 July 2018.**

**This search was adapted for the other databases**

1 (anaesthetist* or anesthetist* or audiologist* or cardiologist* or chiropodist* or clinician* or consultant* or cadet* or counsellor* or dentist* or dermatologist* or dietician* or Doctor* or GP or GPs or gynaecologist* or gynecologist* or matron* or midwife or midwives or neurologist* or nurse* or nutritionist* or obstetrician* or oncologist* or optometrist* or orthodontist* or orthoptist* or orthotist* or osteopath* or paediatrician* or pediatrician* or paramedic* or pathologist* or pharmacist* or phlebotomist* or physician* or physiologist* or physiotherapist* or podiatrist* or practice manager* or practice staff or practitioner* or prosthetist* or psychiatrist* or psychologist* or psychotherapist* or radiographer* or radiologist* or registrar* or rheumatologist* or surgeon* or therapist* or urologist* or anesthesiologist* or prescriber* or sonographer*).ab,ti. (1380041)

2 exp Health Personnel/ (462248)

3 exp CONSULTANTS/ (6476)

4 ((ambulance or associate or audiology or cardiology or chiropody or clinical or dental or dermatology or family or gynaecology or gynecology or health or healthcare or "health care" or hospital or house or medical or midwifery or neurology or nursing or nutrition or obstetrics or oncology or optometry or orthodontic or paediatric* or pediatric* or pathology or pharmacy or physiology or physiotherapy or podiatry or psychiatry or psychology or "public health" or radiolog* or rheumatology or surgical or therapy or trainee or urology or respiratory or magnetic resonance imaging) adj2 (assistant* or cadet* or director* or manager* or officer* or personnel or practice or practitioner* or professional* or provider or receptionist* or resident* or scientist* or secretar* or specialist* or staff or technician* or technologist or visitor* or worker*)).ab,ti. (499319)

5 1 or 2 or 3 or 4 (1895700)

6 benchmark*.ab,ti. (30808)

7 ((audit or monitoring or peer or performance or data or individualised or individualized or web or personalised or personalized or compar* or team or practitioner or practice or clinical or social) adj2 feedback).ab,ti. (6421)

8 FEEDBACK, PSYCHOLOGICAL/ (3122)

9 ((social or descriptive or peer or subjective) adj2 (norm or norms)).ab,ti. (5654)

10 social influence.ab,ti. (1497)

11 Social Norms/ (747)

12 BENCHMARKING/ (12165)

13 ((social or peer*) adj2 comparison*).ab,ti. (1649)

14 social competition.ab,ti. (109)

15 social proof.ab,ti. (17)

16 image motivation.ab,ti. (8)

17 warm glow.ab,ti. (27)

18 ((social or verbal or non-verbal or nonverbal or non verbal) adj2 (incentive or incentives or reward or rewards)).ab,ti. (637)

19 positive reinforcement.ab,ti. (1439)

20 "congratul*".ab,ti. (505)

21 praise.ab,ti. (1720)

22 commendation.ab,ti. (68)

23 Reinforcement, Social/ (1036)

24 credible source.ab,ti. (100)

25 Peer Influence/ (273)

26 theory of planned behavio?r.ab,ti. (2518)

27 theory of reasoned action.ab,ti. (453)

28 theoretical domains framework.ab,ti. (249)

29 social cognitive theory.ab,ti. (1344)

30 "theory of normative social behavio?r*".ab,ti. (21)

31 6 or 7 or 8 or 9 or 10 or 11 or 12 or 13 or 14 or 15 or 16 or 17 or 18 or 19 or 20 or 21 or 22 or 23 or 24 or 25 or 26 or 27 or 28 or 29 or 30 (64961)

32 randomized controlled trial.pt. (463949)

33 controlled clinical trial.pt. (92495)

34 "randomiz*".ab,ti. (447770)

35 "randomis*".ab,ti. (89649)

36 placebo.ab. (187269)

37 Clinical Trials as Topic/ (184102)

38 randomly.ab,ti. (288777)

39 trial.ti. (180410)

40 RCT.ab,ti. (15807)

41 32 or 33 or 34 or 35 or 36 or 37 or 38 or 39 or 40 (1188749)

42 exp Animals/ (21630632)

43 Humans/ (17157286)

44 42 not (42 and 43) (4473346)

45 41 not 44 (1094500)

46 5 and 31 and 45 (1486)

Appendix 2 - Data Extraction Form

**Stage One Extraction**

| Section | Field | Notes | Codes |
| --- | --- | --- | --- |
| Identification | Setting |  | 1 Primary (GP and GP practice nurses)  2 Hospital – inpatient,  3 Hospital – outpatient  4 mental health  5 community (district nursing, pharmacy, opticians, podiatry)  6 care/nursing home  7 other  If other add free text |
|  | Country | Free text |  |
| Methods | Design | For stepped wedge choose ‘other’ and enter ‘stepped wedge’  Most trials in this review can be considered cluster randomised – and the most important bit of information is the unit of allocation which is captured elsewhere: however we need to be consistent. For a trial where the unit of allocation is the healthcare worker and data is collected at a patient level, we should class the trial as ‘cluster randomised’ |  |
|  | Unit of allocation |  |  |
|  | Primary outcome | Describe selected behaviour compliance outcome |  |
|  | Secondary outcomes | Describe selected patient outcome |  |
|  | Time points | List all reported, including baseline, in months since randomisation (for compliance with target behaviour) |  |
|  | Statistical analysis | Brief description of statistical analysis, including any adjustment for clustering. Note any problems/errors. |  |

| Population | Target behaviour (short summary) |  | 1 Prescribing,  2 Handwashing  3 test ordering,  4 referrals  5 other  6 unclear  If other add free text after the number |
| --- | --- | --- | --- |
|  | Target behaviour (full) | Add free text description, as described in the paper e.g. ‘Prescribing of antibiotics’ |  |
|  | Total number randomised | Report number of patients and number of healthcare workers |  |
|  | Type of healthcare worker | The profession of the person who was the target of the intervention. | 1 Nurse  2 Doctor – GP,  3 Doctor – Secondary care  4 Pharmacist  5 AHP,  6 Other  If other add free text after the number |

| Interventions | Number of participants randomised to group | Both patients and healthcare workers where relevant |  |
| --- | --- | --- | --- |
|  | Number of clusters randomised to group | Only cluster RCTs |  |
|  | Description | Copy and paste ALL details given by the authors about the intervention. Please include details from any of the study papers.  Flag or paste link(s) to supplementary material(s), where appropriate |  |
|  | Type of control |  | 6 variation of SN  7 usual practice  8 attention control  9 concomitant intervention  10 unsure  11 other (add details) |

| Outcomes |  | See note below on extracting outcomes.  Use ‘add note’ to add extra information e.g. ICC, adjusted values  Choose time point closest to 6 months. |  |
| --- | --- | --- | --- |
| Quality Assessment |  | See Cochrane Handbook Chapter 8  Rate risk of bias (note 2 below) in relation to compliance with target behaviour  See also section 16.3.2 of the Cochrane Handbook ‘Assessing risk of bias in cluster-randomised trials’. | Please use ‘other sources of bias’ to make a judgement on methods of analysis in the case of cluster RCTs. |

**Extracting outcome data: SOCIAL**

Aim: Standardised mean difference and standard error (adjusted for clustering if necessary).

|  | What to extract | Aim |
| --- | --- | --- |
| Scenario 1  Individually randomised  Numerical outcome | Extract mean and standard deviation by group | Calculate standardised mean difference (SMD) and standard error in STATA |
| Scenario 2  Individually randomised  Binary outcome | Extract number having event and totals by group | 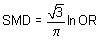  Standard error SMD = $\frac{\surd3}{\pi}\times$ standard error log OR |
| Scenario 3  Cluster randomised  Numerical outcome | Extract mean and standard deviation by group  Extract detail required to calculate ‘design effect’ – e.g. ICC, adjusted and unadjusted standard errors | Calculate standardised mean difference and standard error in STATA  Multiply standard error by the variance inflation factor VIF |
| Scenario 4  Cluster randomised  Binary outcome | Extract number having event and totals by group  Extract detail required to calculate ‘design effect’ – e.g. ICC, adjusted and unadjusted standard errors | Calculate SMD and standard error as above  Multiply standard error by the VIF |

Design effect = 1 + (M-1) ICC where M is average cluster size

Variance inflation factor = square root of design effect

Where ICC not given either (a) use ICC from a similar study(b) use ratio of adjusted/unadjusted standard errors to estimate the variance inflation factor (VIF)

(See Section 16.3.6 of Cochrane Handbook)

Notes:

1. Where possible we want final scores rather than change from baseline.
2. A trial may be described as cluster randomised but have used a summary statistic for each cluster in the analysis: e.g. mean % of patient referrals per GP – in this case there may be no adjustment required (n is number of GPs) and we can class as individually randomised (however we should record both number of patients and number of healthcare workers.
3. Please highlight outcome data (either on paper copy or electronically in case we need to go back for extra information).
4. It should be possible to calculate adjusted standard errors for SMD directly from adjusted standard errors for mean differences or odds ratios…. I just can’t work out a straightforward way. Any ideas welcome.

**Stage Two Extraction**

SN = Social norm intervention

Use -1 throughout for not applicable and -2 for not reported

| **TRIAL INFORMATION** | 1. Does inclusion criteria target participants based on low target performance? |  | 0 No  1 yes  9 unclear |
| --- | --- | --- | --- |

Only code arms that have social norms as part of the intervention: code any arms without SN as -1 throughout

| **INTERVENTION INFORMATION** | 2. Frequency and intensity of intervention | Mixed total of all the times that any social norm element of the intervention was delivered. | 1 Once only  2 Twice  3 More than twice  9 unclear/not reported |
| --- | --- | --- | --- |
|  | 3. Format | Focus on the social norm elements. | 1 Face to face meeting  2 Email,  3 Written (paper),  4 Integrated computerised (part of existing system),  5 Separate computerised (e.g. website),  6 other (add free text)  7. Mixed  9 Unclear/not reported |
|  | 4. Source of the intervention (i.e. the person delivering the intervention) | Focus on the social norm elements.  If the source is unclear, err on the side of interpreting this as the investigator. | 1 Peer  2 Investigator  3 Employer  4. professional body  5. Supervisor or senior colleague  6 Patient  7 *Credible source*  8 Other (add free text)  9 Unclear/not reported |
|  | 5. Is the person delivering the intervention internal or external to the target person's organisation? | Focus on the social norm elements.  If the source is the investigator, assume this is external unless says otherwise | 1 Internal  2 External  9 Unclear/not reported |
|  | 6. Reference group/person | Group/person used as a) the comparison (*social comparison*); or b) source of approval (other BCTs).  If the intervention includes more than one type of social norm and the reference groups are different, code as ‘multiple’.  If researchers intervene with one person and then that person intervenes with HCPs, code as that person.  PROTOCOL | 1 Peer  2 Professional body  3 Senior person (e.g. manager, policy maker, commissioner)  4 Patient(s)  5 other 6 Multiple  9 Unclear/not reported  If other add free text |

| Section | Field | Notes | Codes (delete as appropriate) |
| --- | --- | --- | --- |
| Intervention | Does the author explicitly describe this as SN? |  | 0 No  1 yes |
|  | Direction of change in behaviour that is desired |  | 1 Increase  2 decrease  3 maintenance  4 unclear |

**Appendix 3 – Behaviour Change Techniques (BCTs) Extraction Form**

| **Target population:** |  | | | |
| --- | --- | --- | --- | --- |
| **Target behaviour:** |  | | | |
| **Arm** | | ***Initials* BCTs** | ***Initials* BCTs** | **Final agreed BCTs** |
| **Control**  ***(Name)*** | |  |  |  |
| *If guidelines present* | | **Guideline unspecified (delete as appropriate):** Yes/No (list BCTs below)  **Guideline BCTs (list)**: | **Guideline unspecified (delete as appropriate):** Yes/No (list BCTs below) | **Guideline unspecified (delete as appropriate):** Yes/No (list BCTs below) |
| **Intervention 1**  ***(Name)*** | |  |  |  |
| *If guidelines present* | | **Guideline unspecified (delete as appropriate):** Yes/No (list BCTs below)  **Guideline BCTs (list)**: | **Guideline unspecified (delete as appropriate):** Yes/No (list BCTs below) | **Guideline unspecified (delete as appropriate):** Yes/No (list BCTs below) |

| **Section** | **Field** | **Notes** | **Codes (delete as appropriate)** |
| --- | --- | --- | --- |
| **Methods** | **Type of comparison** | **Type 1**  SN v any control  This could be social norm v current practice, social norm v nothing, social norm v a non-social norms BCT  **Type 2**  SN + X v X  This is any trial where it is the social norms element that is being tested  E.g. *social comparison* with audit v audit alone  E.g. *credible source* with education v education alone  **Type 3**  SN + X v any control  This is any trial where the social norms element packaged with other BCTs is being tested  E.g. social norms with education and audit v usual practice  E.g. multifaceted intervention with social norms element v nothing  **Type 4**  Either two different social norms interventions or the same social norms interventions packaged in different ways  E.g. *social comparison* v *social reward*  *Social comparison* via email v *social comparison* face-to-face    **Type 5**  The *social comparison* element is identical in each arm, but the other interventions differ  e.g. *social comparison* with reminders v *social comparison* without reminders  e.g. *credible source* with education v *credible source* without education. | 1 SN v any control  2 SN + X v X  3 SN + X v any control  4 SN type A v SN type B  5 SN + X v SN + Y |
| **Interventions** | **Does the author explicitly describe this as SN?** |  | 0 No  1 yes |
|  | **Is SN a substantial part (type 3 above)?** |  | 0 No  1 yes  2 unsure |
|  | **Concomitant intervention (type 2, 3 or 5 above)** |  | 0 No  1 yes |
|  | **Direction of change in behaviour that is desired** |  | 1 Increase  2 decrease  3 maintenance  4 unclear |

**Appendix 4 – Inter-rater agreement for BCT Coding (PABAK)**

PABAK agreement between trained coder pairs for BCTs present at least once in the included unique arms*

| **Behaviour Change Technique (BCT) Label (ordered according to frequency)** | ***N* where BCT was present across the included unique arms*** | **PABAK†** | **Confidence Intervals** |
| --- | --- | --- | --- |
| **Social norm BCTS** |  |  |  |
| 6.2. *Social comparison* | 117 | 0.92 | 0.87, 0.97 |
| 9.1. *Credible source* | 24 | 0.83 | 0.76, 0.91 |
| 6.3. *Information about others’ approval* | 10 | 0.96 | 0.92, 1.00 |
| 10.4. *Social reward* | 5 | 0.97 | 0.93, 1.00 |
| 10.5. *Social incentive* | 1 | 0.99 | 0.97, 1.00 |
| **All other identified BCTs (present at least once)** |  |  |  |
| 2.2. Feedback on behaviour | 130 | 0.79 | 0.71, 0.87 |
| 4.1. Instruction on how to perform the behaviour | 48 | 0.65 | 0.55, 0.75 |
| 3.1. Social support (unspecified) | 41 | 0.83 | 0.75, 0.90 |
| 7.1. Prompts/cues | 29 | 0.79 | 0.71, 0.87 |
| 5.1. Information about health consequences | 26 | 0.80 | 0.72, 0.88 |
| 1.2. Problem solving | 17 | 0.86 | 0.79, 0.93 |
| 12.5. Adding objects to the environment | 14 | 0.88 | 0.82, 0.94 |
| 1.1 Goal setting (behaviour) | 10 | 0.91 | 0.85, 0.97 |
| 2.7. Feedback on outcome(s) of behaviour | 10 | 0.87 | 0.80, 0.93 |
| 6.1. Demonstration of the behaviour | 6 | 0.97 | 0.93, 1.00 |
| 8.1. Behavioural practice/rehearsal | 5 | 0.98 | 0.96, 1.00 |
| 1.3. Goal setting (outcome) | 4 | 0.95 | 0.91, 0.99 |
| 1.4. Action planning | 4 | 0.91 | 0.86, 0.97 |
| 2.3. Self-monitoring of behaviour | 4 | 0.93 | 0.88, 0.98 |
| 12.1. Restructuring the social environment | 3 | 0.98 | 0.96, 1.00 |
| 12.2. Restructuring the social environment | 3 | 0.97 | 0.93, 1.00 |
| 5.3. Information about social and environmental consequences | 3 | 0.98 | 0.96, 1.00 |
| 1.7. Review outcome goal(s) | 2 | 0.97 | 0.93, 1.00 |
| 10.1. Material incentive (behaviour) | 2 | 0.98 | 0.96, 1.00 |
| 2.1. Monitoring of behaviour by others without feedback | 2 | 0.96 | 0.92, 0.99 |
| 9.2. Pros and cons | 2 | 0.97 | 0.94, 1.00 |
| 1.9. Commitment | 1 | 0.99 | 0.97, 1.00 |
| 10.3. Non-specific reward | 1 | 0.98 | 0.96, 1.00 |
| 2.5. Monitoring of behaviour without feedback | 1 | Ratings do not vary | |
| 3.2. Social support (practical) | 1 | 0.97 | 0.93, 1.00 |
| 8.2. Behaviour substitution | 1 | 0.99 | 0.97, 1.00 |

*229 unique included arms

†To calculate PABAK, the KAPPAETC module in Stata was used to produce the Brennan-Prediger statistic

**Appendix 5 - Included Study References**

1. Aspy CB, Enright M Fau - Halstead L, Halstead L Fau - Mold JW, Mold JW. Improving mammography screening using best practices and practice enhancement assistants: an Oklahoma Physicians Resource/Research Network (OKPRN) study. Journal of the American Board of Family Medicine. 2008;21:326-33.

2. Awad AI, Eltayeb Ib Fau - Baraka OZ, Baraka OZ. Changing antibiotics prescribing practices in health centers of Khartoum State, Sudan. Eur J Clin Pharmacol 62:135-42.

3. Baker R, Farooqi A, Tait C, Walsh S. Randomised controlled trial of reminders to enhance the impact of audit in general practice on management of patients who use benzodiazepines. BMJ Quality & Safety 1997;6(1):14-8.

4. Barnett KN, Bennie M, Treweek S, Robertson C, Petrie DJ, Ritchie LD, et al. Effective Feedback to Improve Primary Care Prescribing Safety (EFIPPS) a pragmatic three-arm cluster randomised trial: designing the intervention (ClinicalTrials.gov registration NCT01602705). Implementation Science. 2014;9(1).

5. Baskerville NB, Hogg W Fau - Lemelin J, Lemelin J. Process evaluation of a tailored multifaceted approach to changing family physician practice patterns improving preventive care. The Journal of family practice 2001;50:242-9.

6. Beck CA, Richard H, Tu JV, Pilote L. Administrative Data Feedback for Effective Cardiac Treatment. Jama. 2005;294(3):309-17.

7. Beidas RS, Becker-Haimes EM, Adams DR, Skriner L, Stewart RE, Wolk CB, et al. Feasibility and acceptability of two incentive-based implementation strategies for mental health therapists implementing cognitive-behavioral therapy: a pilot study to inform a randomized controlled trial. Implementation Science. 2017;12(1).

8. Bentz CJ, Bayley Kb Fau - Bonin KE, Bonin Ke Fau - Fleming L, Fleming L Fau - Hollis JF, Hollis Jf Fau - Hunt JS, Hunt Js Fau - LeBlanc B, et al. Provider feedback to improve 5A's tobacco cessation in primary care: a cluster randomized clinical trial. Nicotine & Tobacco Research 2007. 2007;9:341-9.

9. Bhatia RS, Ivers NM, Yin XC, Myers D, Nesbitt GC, Edwards J, et al. Improving the Appropriate Use of Transthoracic Echocardiography. Journal of the American College of Cardiology. 2017;70(9):1135-44.

10. Bhattacharyya O, Harris S, Zwarenstein M, Barnsley J. Controlled trial of an intervention to improve cholesterol management in diabetes patients in remote Aboriginal communities. International journal of circumpolar health 2010;69(4):333-43.

11. Billue KL, Safford MM, Salanitro AH, Houston TK, Curry W, Kim Y, et al. Medication intensification in diabetes in rural primary care: a cluster-randomised effectiveness trial. BMJ Open. 2012;2(5):e000959.

12. Boet SA-OX, Bryson GL, Taljaard M, Pigford AA, McIsaac DI, Brehaut J, et al. Effect of audit and feedback on physicians' intraoperative temperature management and patient outcomes: a three-arm cluster randomized-controlled trial comparing benchmarked and ranked feedback. Canadian Journal of Anesthesia/Journal canadien d'anesthésie. 2018;65:1196-209.

13. Braybrook S, Walker R. Influencing prescribing in primary care: a comparison of two different prescribing feedback methods. Journal of clinical pharmacy and therapeutics 1996;21(4):247-54.

14. Brinkman WB, Geraghty SR, Lanphear BP, Khoury JC, Gonzalez Del Rey JA, Dewitt TG, et al. Effect of Multisource Feedback on Resident Communication Skills and Professionalism. Archives of Pediatrics & Adolescent Medicine. 2007;161(1):44.

15. Brown B, Young J, Smith DP, Kneebone AB, Brooks AJ, Egger S, et al. A multidisciplinary team-oriented intervention to increase guideline recommended care for high-risk prostate cancer: A stepped-wedge cluster randomised implementation trial. Implementation Science. 2018;13(1).

16. Buntinx F, Knottnerus J, Crebolder H, Seegers T, Essed GGM, Schouten H. Does Feedback Improve the Quality of Cervical Smears? A Randomized Controlled Trial. The British journal of general practice : the journal of the Royal College of General Practitioners. 1993;43:194-8.

17. Butler CC, Simpson SA, Dunstan F, Rollnick S, Cohen D, Gillespie D, et al. Effectiveness of multifaceted educational programme to reduce antibiotic dispensing in primary care: practice based randomised controlled trial. BMJ. 2012;344(feb02 1):d8173-d.

18. Carney PA, Abraham L, Cook A, Feig SA, Sickles EA, Miglioretti DL, et al. Impact of an Educational Intervention Designed to Reduce Unnecessary Recall during Screening Mammography. Academic radiology. 2012;19(9):1114-20.

19. Cheater FM, Baker R, Reddish S, Spiers N, Wailoo A, Gillies C, et al. Cluster Randomized Controlled Trial of the Effectiveness of Audit and Feedback and Educational Outreach on Improving Nursing Practice and Patient Outcomes. Medical Care. 2006;44(6):542-51.

20. Colón-Emeric CS, Lyles KW, House P, Levine DA, Schenck AP, Allison J, et al. Randomized Trial to Improve Fracture Prevention in Nursing Home Residents. American Journal of Medicine 2007;120(10):886-92.

21. Curtis JR, Olivieri J, Allison JJ, Gaffo A, Juarez L, Kovac SH, et al. A group randomized trial to improve safe use of nonsteroidal anti-inflammatory drugs. Am J Manag Care. 2005;11(9):537-43.

22. Curtis JR, Westfall AO, Allison J, Becker A, Melton ME, Freeman A, et al. Challenges in Improving the Quality of Osteoporosis Care for Long-term Glucocorticoid Users. Archives of internal medicine 2007;167(6):591-6.

23. Desveaux L, Gomes T, Tadrous M, Jeffs L, Taljaard M, Rogers J, et al. Appropriate prescribing in nursing homes demonstration project (APDP) study protocol: pragmatic, cluster-randomized trial and mixed methods process evaluation of an Ontario policy-maker initiative to improve appropriate prescribing of antipsychotics. Implementation Science. 2015;11(1).

24. Eccles M, Steen N, Grimshaw J, Thomas L, McNamee P, Soutter J, et al. Effect of audit and feedback, and reminder messages on primary-care radiology referrals: a randomised trial. The Lancet. 2001;357(9266):1406-9.

25. Elouafkaoui P, Young L, Newlands R, Duncan EM, Elders A, Clarkson JA-O, et al. An Audit and Feedback Intervention for Reducing Antibiotic Prescribing in General Dental Practice: The RAPiD Cluster Randomised Controlled Trial. PLoS Medicine 2016;13(1549-1676 (Electronic)).

26. Eltayeb IB. Changing the prescribing patterns of sexually transmitted infections in the White Nile Region of Sudan. Sexually transmitted infections 2005;81(5):426-7.

27. Ferguson JTB. Use of Continuous Quality Improvement to Increase Use of Process Measures in Patients Undergoing Coronary Artery Bypass Graft Surgery: A Randomized Controlled Trial. Journal of the American Medical Association 2003;290(1):49-56.

28. Fiks AG, Mayne Sl Fau - Michel JJ, Michel Jj Fau - Miller J, Miller J Fau - Abraham M, Abraham M Fau - Suh A, Suh A Fau - Jawad AF, et al. Distance-Learning, ADHD Quality Improvement in Primary Care: A Cluster-Randomized Trial. Journal of developmental and behavioral pediatrics : JDBP 2017;38(1536-7312 (Electronic)):573-83.

29. Foster JM, Hoskins G, Smith B, Lee AJ, Price D, Pinnock H. Practice development plans to improve the primary care management of acute asthma: randomised controlled trial. BMC family practice 2007;8(1):23.

30. Foy R, Penney GC, Grimshaw JM, Ramsay CR, Walker AE, MacLennan G, et al. A randomised controlled trial of a tailored multifaceted strategy to promote implementation of a clinical guideline on induced abortion care. BJOG: An International Journal of Obstetrics and Gynaecology 2004;111(7):726-33.

31. French SD, McKenzie JE, O'Connor DA, Grimshaw JM, Mortimer D, Francis JJ, et al. Evaluation of a Theory-Informed Implementation Intervention for the Management of Acute Low Back Pain in General Medical Practice: The IMPLEMENT Cluster Randomised Trial. PLoS ONE. 2013;8(6):e65471.

32. Fuller C, Michie S, Savage J, McAteer J, Besser S, Charlett A, et al. The Feedback Intervention Trial (FIT) — Improving Hand-Hygiene Compliance in UK Healthcare Workers: A Stepped Wedge Cluster Randomised Controlled Trial. PLoS ONE. 2012;7(10):e41617.

33. Gascón Cánovas JJ, Saturno Hernández PJ, Antón Botella JJ. Effectiveness of internal quality assurance programmes in improving clinical practice and reducing costs. Journal of evaluation in clinical practice 2009;15(5):813-9.

34. Gerber JS, Prasad Pa Fau - Fiks AG, Fiks Ag Fau - Localio AR, Localio Ar Fau - Grundmeier RW, Grundmeier Rw Fau - Bell LM, Bell Lm Fau - Wasserman RC, et al. Effect of an outpatient antimicrobial stewardship intervention on broad-spectrum antibiotic prescribing by primary care pediatricians: a randomized trial. JAMA - Journal of the American Medical Association. 2013;309:2345-52.

35. Gjelstad S, Fetveit A Fau - Straand J, Straand J Fau - Dalen I, Dalen I Fau - Rognstad S, Rognstad S Fau - Lindbaek M, Lindbaek M. Can antibiotic prescriptions in respiratory tract infections be improved? A cluster-randomized educational intervention in general practice--the Prescription Peer Academic Detailing (Rx-PAD) Study [NCT00272155]. BMC health services research 2006;6(1472-6963 (Electronic)):75.

36. Goff DC, Jr., Gu L Fau - Cantley LK, Cantley Lk Fau - Parker DG, Parker Dg Fau - Cohen SJ, Cohen SJ. Enhancing the quality of care for patients with coronary heart disease: the design and baseline results of the hastening the effective application of research through technology (HEART) trial. American journal of managed care 2002;8.

37. Guadagnoli E, Soumerai Sb Fau - Gurwitz JH, Gurwitz Jh Fau - Borbas C, Borbas C Fau - Shapiro CL, Shapiro Cl Fau - Weeks JC, Weeks Jc Fau - Morris N, et al. Improving discussion of surgical treatment options for patients with breast cancer: local medical opinion leaders versus audit and performance feedback. Breast Cancer Research and Treatment 2000(61):171-5.

38. Guldberg TL, Vedsted P Fau - Kristensen JK, Kristensen Jk Fau - Lauritzen T, Lauritzen T. Improved quality of Type 2 diabetes care following electronic feedback of treatment status to general practitioners: a cluster randomized controlled trial. Diabetic Medicine. 2011;28(3):325-32.

39. Hallsworth M, Chadborn T, Sallis A, Sanders M, Berry D, Greaves F, et al. Provision of social norm feedback to high prescribers of antibiotics in general practice: a pragmatic national randomised controlled trial. The Lancet. 2016;387(10029):1743-52.

40. Hayashino Y, Suzuki H, Yamazaki K, Goto A, Izumi K, Noda M. A cluster randomized trial on the effect of a multifaceted intervention improved the technical quality of diabetes care by primary care physicians: The Japan Diabetes Outcome Intervention Trial-2 (J-DOIT2). Diabetic Medicine. 2016;33(5):599-608.

41. Hayes RP, Baker Dw Fau - Luthi J-C, Luthi Jc Fau - Baggett RL, Baggett Rl Fau - McClellan W, McClellan W Fau - Fitzgerald D, Fitzgerald D Fau - Abrams FR, et al. The effect of external feedback on the management of medicare inpatients with congestive heart failure. American Journal of Medical Quality. 2002;17(6):225-35.

42. Heller RF, D'Este C Fau - Lim LL, Lim Ll Fau - O'Connell RL, O'Connell Rl Fau - Powell H, Powell H. Randomised controlled trial to change the hospital management of unstable angina. Medical journal of Australia. 2001;174(5):217-21.

43. Hemkens LG, Saccilotto R, Reyes SL, Glinz D, Zumbrunn T, Grolimund O, et al. Personalized Prescription Feedback Using Routinely Collected Data to Reduce Antibiotic Use in Primary Care: A Randomized Clinical Trial. JAMA internal medicine. 2017;177(2):176-83.

44. Herbert CP, Wright JM, Maclure M, Wakefield J, Dormuth C, Brett-MacLean P, et al. Better Prescribing Project: a randomized controlled trial of the impact of case-based educational modules and personal prescribing feedback on prescribing for hypertension in primary care. Fam Pract. 2004;21(5):575-81.

45. Horbar JD, Carpenter JH, Buzas J, Soll RF, Suresh G, Bracken MB, et al. Collaborative quality improvement to promote evidence based surfactant for preterm infants: a cluster randomised trial. Bmj. 2004;329(7473):1004.

46. Houston TK, Sadasivam RS, Allison JJ, Ash AS, Ray MN, English TM, et al. Evaluating the QUIT-PRIMO clinical practice ePortal to increase smoker engagement with online cessation interventions: a national hybrid type 2 implementation study. Implementation Science. 2015;10(1):154.

47. Howe A. Detecting psychological distress: can general practitioners improve their own performance? The British journal of general practice : the journal of the Royal College of General Practitioners. 1996;46(408):407-10.

48. Huis A, Schoonhoven L Fau - Grol R, Grol R Fau - Donders R, Donders R Fau - Hulscher M, Hulscher M Fau - van Achterberg T, van Achterberg T. Impact of a team and leaders-directed strategy to improve nurses' adherence to hand hygiene guidelines: a cluster randomised trial. International journal of nursing studies. 2013;50(4):464-74.

49. Hysong SJ, Simpson K Fau - Pietz K, Pietz K Fau - SoRelle R, SoRelle R Fau - Broussard Smitham K, Broussard Smitham K Fau - Petersen LA, Petersen LA. Financial incentives and physician commitment to guideline-recommended hypertension management. The American journal of managed care 2012;18(10):e375.

50. Ivers NM, Tu K, Young J, Francis JJ, Barnsley J, Shah BR, et al. Feedback GAP: pragmatic, cluster-randomized trial of goal setting and action plans to increase the effectiveness of audit and feedback interventions in primary care. Implementation Science. 2013;8(1):142.

51. Katz DA, Muehlenbruch Dr Fau - Brown RL, Brown Rl Fau - Fiore MC, Fiore Mc Fau - Baker TB, Baker TB. Effectiveness of implementing the agency for healthcare research and quality smoking cessation clinical practice guideline: a randomized, controlled trial. Journal of the National Cancer Institute. 2004;96(8):594-603.

52. Kaufman R, Stanworth S, Taljaard M, Yazer M, Reeves C, Delaney M, et al. Trial of Feedback on Blood Use2016. 30A-A p.

53. Kennedy CC, Ioannidis G, Thabane L, Adachi JD, Marr S, Giangregorio LM, et al. Successful knowledge translation intervention in long-term care: final results from the vitamin D and osteoporosis study (ViDOS) pilot cluster randomized controlled trial. Trials. 2015;16(1).

54. Kiefe CI, Allison Jj Fau - Williams OD, Williams Od Fau - Person SD, Person Sd Fau - Weaver MT, Weaver Mt Fau - Weissman NW, Weissman NW. Improving quality improvement using achievable benchmarks for physician feedback: a randomized controlled trial. Jama. 2001;285(22):2871-9.

55. Kim CS, Kristopaitis Rj Fau - Stone E, Stone E Fau - Pelter M, Pelter M Fau - Sandhu M, Sandhu M Fau - Weingarten SR, Weingarten SR. Physician education and report cards: do they make the grade? results from a randomized controlled trial. The American journal of medicine. 1999;107(6):556-60.

56. Koff MD, Brown JR, Marshall EJ, O'Malley AJ, Jensen JT, Heard SO, et al. Frequency of Hand Decontamination of Intraoperative Providers and Reduction of Postoperative Healthcare-Associated Infections: A Randomized Clinical Trial of a Novel Hand Hygiene System. Infection control & hospital epidemiology. 2016;37(8):888-95.

57. Kogan JR, Reynolds Ee Fau - Shea JA, Shea JA. Effectiveness of report cards based on chart audits of residents' adherence to practice guidelines on practice performance: a randomized controlled trial. Teaching and learning in medicine. 2003;15(1):25-30.

58. Lakshminarayan K, Borbas C Fau - McLaughlin B, McLaughlin B Fau - Morris NE, Morris Ne Fau - Vazquez G, Vazquez G Fau - Luepker RV, Luepker Rv Fau - Anderson DC, et al. A cluster-randomized trial to improve stroke care in hospitals. Neurology. 2010;74(20):1634-42.

59. Leviton LC, Goldenberg Rl Fau - Baker CS, Baker Cs Fau - Schwartz RM, Schwartz Rm Fau - Freda MC, Freda Mc Fau - Fish LJ, Fish Lj Fau - Cliver SP, et al. Methods to encourage the use of antenatal corticosteroid therapy for fetal maturation: a randomized controlled trial. Jama. 1999;281(1):46-52.

60. Liddy C, Hogg W Fau - Russell G, Russell G Fau - Wells G, Wells G Fau - Armstrong CD, Armstrong Cd Fau - Akbari A, Akbari A Fau - Dahrouge S, et al. Improved delivery of cardiovascular care (IDOCC) through outreach facilitation: study protocol and implementation details of a cluster randomized controlled trial in primary care. Implementation Science. 2011;6(1):110.

61. Lim WY, Hss AS, Ng LM, John Jasudass SR, Sararaks S, Vengadasalam P, et al. The impact of a prescription review and prescriber feedback system on prescribing practices in primary care clinics: a cluster randomised trial. BMC Family Practice. 2018;19(1).

62. Lomas J, Enkin M Fau - Anderson GM, Anderson Gm Fau - Hannah WJ, Hannah Wj Fau - Vayda E, Vayda E Fau - Singer J, Singer J. Opinion leaders vs audit and feedback to implement practice guidelines. Delivery after previous cesarean section. Jama. 1991;265(17):2202-7.

63. MacLean CD, Littenberg B, Gagnon M, Reardon M, Turner PD, Jordan C. The Vermont Diabetes Information System (VDIS): study design and subject recruitment for a cluster randomized trial of a decision support system in a regional sample of primary care practices. Clin Trials. 2004;1(6):532-44.

64. Mayne SL, duRivage NE, Feemster KA, Localio AR, Grundmeier RW, Fiks AG. Effect of decision support on missed opportunities for human papillomavirus vaccination. Effect of decision support on missed opportunities for human papillomavirus vaccination. 2014;47(6):734-44.

65. McClellan WM, Millman L Fau - Presley R, Presley R Fau - Couzins J, Couzins J Fau - Flanders WD, Flanders WD. Improved diabetes care by primary care physicians: results of a group-randomized evaluation of the Medicare Health Care Quality Improvement Program (HCQIP). Journal of clinical epidemiology. 2003;56(12):1210-7.

66. McCluskey A, Ada L, Kelly PJ, Middleton S, Goodall S, Grimshaw JM, et al. A behavior change program to increase outings delivered during therapy to stroke survivors by community rehabilitation teams: The Out-and-About trial. International Journal of Stroke. 2016;11(4):425-37.

67. McPhee SJ, Bird Ja Fau - Jenkins CN, Jenkins Cn Fau - Fordham D, Fordham D. Promoting cancer screening. A randomized, controlled trial of three interventions. Archives of internal medicine. 1989;149(8):1866-72.

68. Meeker D, Linder JA, Fox CR, Friedberg MW, Persell SD, Goldstein NJ, et al. Effect of Behavioral Interventions on Inappropriate Antibiotic Prescribing Among Primary Care Practices. JAMA. 2016;315(6):562.

69. Metlay JP, Camargo Ca Jr Fau - MacKenzie T, MacKenzie T Fau - McCulloch C, McCulloch C Fau - Maselli J, Maselli J Fau - Levin SK, Levin Sk Fau - Kersey A, et al. Cluster-randomized trial to improve antibiotic use for adults with acute respiratory infections treated in emergency departments. Annals of Emergency Medicine. 2007;50(3):221-30.

70. Mold JW, Aspy Ca Fau - Nagykaldi Z, Nagykaldi Z. Implementation of evidence-based preventive services delivery processes in primary care: an Oklahoma Physicians Resource/Research Network (OKPRN) study. The Journal of the American Board of Family Medicine 2008;21(4):334-44.

71. Mold JW, Fox C Fau - Wisniewski A, Wisniewski A Fau - Lipman PD, Lipman Pd Fau - Krauss MR, Krauss Mr Fau - Harris DR, Harris Dr Fau - Aspy C, et al. Implementing asthma guidelines using practice facilitation and local learning collaboratives: a randomized controlled trial. The Annals of Family Medicine. 2014;12(3):233-40.

72. Morrison LJ, Brooks Sc Fau - Dainty KN, Dainty Kn Fau - Dorian P, Dorian P Fau - Needham DM, Needham Dm Fau - Ferguson ND, Ferguson Nd Fau - Rubenfeld GD, et al. Improving use of targeted temperature management after out-of-hospital cardiac arrest: a stepped wedge cluster randomized controlled trial. Critical Care Medicine. 2015;43(5):954-64.

73. Mourad SM, Hermens RPMG, Liefers J, Akkermans RP, Zielhuis GA, Adang E, et al. A multi-faceted strategy to improve the use of national fertility guidelines; a cluster-randomized controlled trial. Human Reproduction. 2011;26(4):817-26.

74. O'Connell DL, Henry D Fau - Tomlins R, Tomlins R. Randomised controlled trial of effect of feedback on general practitioners' prescribing in Australia. Bmj. 1999;318(7182):507-11.

75. O'Connor PJ, Sperl-Hillen J Fau - Johnson PE, Johnson Pe Fau - Rush WA, Rush Wa Fau - Crain AL, Crain AL. Customized feedback to patients and providers failed to improve safety or quality of diabetes care: a randomized trial. Diabetes Care. 2009;32(7):1158-63.

76. Patel MS, Kurtzman GW, Kannan S, Small DS, Morris A, Honeywell S, et al. Effect of an Automated Patient Dashboard Using Active Choice and Peer Comparison Performance Feedback to Physicians on Statin Prescribing. JAMA Network Open. 2018;1(3):e180818.

77. Peiris D, Usherwood T, Panaretto K, Harris M, Hunt J, Redfern J, et al. Effect of a computer-guided, quality improvement program for cardiovascular disease risk management in primary health care: the treatment of cardiovascular risk using electronic decision support cluster-randomized trial. Circulation: Cardiovascular Quality and Outcomes. 2015;8(1):87-95.

78. Persell SD, Doctor JN, Friedberg MW, Meeker D, Friesema E, Cooper A, et al. Behavioral interventions to reduce inappropriate antibiotic prescribing: a randomized pilot trial. BMC Infectious Diseases. 2016;16(1):373.

79. Pimlott NJ, Hux Je Fau - Wilson LM, Wilson Lm Fau - Kahan M, Kahan M Fau - Li C, Li C Fau - Rosser WW, Rosser WW. Educating physicians to reduce benzodiazepine use by elderly patients: a randomized controlled trial. Cmaj. 2003;168(7):835-9.

80. Pope J, Thorne C Fau - Cividino A, Cividino A Fau - Lucas K, Lucas K. Effect of rheumatologist education on systematic measurements and treatment decisions in rheumatoid arthritis: the metrix study. The Journal of Rheumatology. 2012;39(12):2247-52.

81. Quinley JC, Shih A. Improving Physician Coverage of Pneumococcal Vaccine: A Randomized Trial of a Telephone Intervention. Journal of Community Health. 2004;29(2):103-15.

82. Raasch BA, Hays R Fau - Buettner PG, Buettner PG. An educational intervention to improve diagnosis and management of suspicious skin lesions. Journal of Continuing Education in the Health professions. 2000;20(1):39-51.

83. Raja AS, Ip IK, Dunne RM, Schuur JD, Mills AM, Khorasani R. Effects of Performance Feedback Reports on Adherence to Evidence-Based Guidelines in Use of CT for Evaluation of Pulmonary Embolism in the Emergency Department: A Randomized Trial. American Journal of Roentgenology. 2015;205(5):936-40.

84. Rask K, Kohler SA, Wells K, Williams J, Diamond C, editors. Performance Improvement Interventions to Improve Delivery of Screening Services in Diabetes Care2001.

85. Sandbaek A, Kragstrup J. Randomized controlled trial of the effect of medical audit on AIDS prevention in general practice. Family practice. 1999;16(5):510-4.

86. Sauaia A, Ralston D, Schluter WW, Marciniak TA, Havranek EP, Dunn TR. Influencing Care in Acute Myocardial Infarction: A Randomized Trial Comparing 2 Types of Intervention. American Journal of Medical Quality. 2000;15(5):197-206.

87. Schneider A, Wensing M Fau - Biessecker K, Biessecker K Fau - Quinzler R, Quinzler R Fau - Kaufmann-Kolle P, Kaufmann-Kolle P Fau - Szecsenyi J, Szecsenyi J. Impact of quality circles for improvement of asthma care: results of a randomized controlled trial. Journal of evaluation in clinical practice. 2008;14(2):185-90.

88. Soleymani F, Rashidian A Fau - Dinarvand R, Dinarvand R Fau - Kebriaeezade A, Kebriaeezade A Fau - Hosseini M, Hosseini M Fau - Abdollahi M, Abdollahi M. Assessing the effectiveness and cost-effectiveness of audit and feedback on physician's prescribing indicators: study protocol of a randomized controlled trial with economic evaluation. DARU Journal of Pharmaceutical Sciences. 2012;20(1):88.

89. Søndergaard J, Andersen M Fau - Støvring H, Støvring H Fau - Kragstrup J, Kragstrup J. Mailed prescriber feedback in addition to a clinical guideline has no impact: a randomised, controlled trial. Scandinavian journal of primary health care. 2003;21(1):47-51.

90. Søndergaard J, Andersen M Fau - Vach K, Vach K Fau - Kragstrup J, Kragstrup J Fau - Maclure M, Maclure M Fau - Gram LF, Gram LF. Detailed postal feedback about prescribing to asthma patients combined with a guideline statement showed no impact: a randomised controlled trial. European journal of clinical pharmacology. 2002;58(1):127-32.

91. Soumerai SB, McLaughlin Tj Fau - Gurwitz JH, Gurwitz Jh Fau - Guadagnoli E, Guadagnoli E Fau - Hauptman PJ, Hauptman Pj Fau - Borbas C, Borbas C Fau - Morris N, et al. Effect of local medical opinion leaders on quality of care for acute myocardial infarction: a randomized controlled trial. Jama. 1998;279(17):13-58-63.

92. Stewardson AJ, Sax H, Gayet-Ageron A, Touveneau S, Longtin Y, Zingg W, et al. Enhanced performance feedback and patient participation to improve hand hygiene compliance of health-care workers in the setting of established multimodal promotion: a single-centre, cluster randomised controlled trial. The Lancet Infectious Diseases. 2016;16(12):1345-55.

93. Thomas RE, Croal BL, Ramsay C, Eccles M, Grimshaw J. Effect of enhanced feedback and brief educational reminder messages on laboratory test requesting in primary care: a cluster randomised trial. Lancet. 2006;367(9527):1990-6.

94. Tjia J, Field T, Mazor K, Lemay CA, Kanaan AO, Donovan JL, et al. Dissemination of Evidence-Based Antipsychotic Prescribing Guidelines to Nursing Homes: A Cluster Randomized Trial. Journal of the American Geriatrics Society. 2015;63(7):1289-98.

95. Trietsch J, van Steenkiste B, Grol R, Winkens B, Ulenkate H, Metsemakers J, et al. Effect of audit and feedback with peer review on general practitioners' prescribing and test ordering performance: a cluster-randomized controlled trial. BMC family practice. 2017;18(1):53.

96. van Bruggen R, Gorter KJ, Stolk RP, Verhoeven RP, Rutten GEHM. Implementation of locally adapted guidelines on type 2 diabetes. Family Practice. 2008;25(6):430-7.

97. Vellinga A, Galvin S, Duane S, Callan A, Bennett K, Cormican M, et al. Intervention to improve the quality of antimicrobial prescribing for urinary tract infection: a cluster randomized trial. Canadian Medical Association Journal. 2016;188(2):108-15.

98. Verstappen WH, van der Weijden T Fau - Sijbrandij J, Sijbrandij J Fau - Smeele I, Smeele I Fau - Hermsen J, Hermsen J Fau - Grimshaw J, Grimshaw J Fau - Grol RPTM, et al. Effect of a practice-based strategy on test ordering performance of primary care physicians: a randomized trial. Jama. 2003;289(18):2407-12.

99. Voorn VMA, Marang-van de Mheen PJ, van der Hout A, Hofstede SN, So-Osman C, van den Akker-van Marle ME, et al. The effectiveness of a de-implementation strategy to reduce low-value blood management techniques in primary hip and knee arthroplasty: a pragmatic cluster-randomized controlled trial. Implementation Science. 2017;12(1):72.

100. Wang Y, Li Z, Zhao X, Wang C, Wang X, Wang D, et al. Effect of a Multifaceted Quality Improvement Intervention on Hospital Personnel Adherence to Performance Measures in Patients With Acute Ischemic Stroke in China: A Randomized Clinical Trial. Jama. 2018;320(3):245-54.

101. Watkins RS, Moran WP. Competency-based learning: the impact of targeted resident education and feedback on Pap smear adequacy rates. J Gen Interen Med. 2004;19:545-8.

102. Weitzman S, Greenfield S Fau - Billimek J, Billimek J Fau - Hava T, Hava T Fau - Schvartzman P, Schvartzman P Fau - Yehiel E, Yehiel E Fau - Tandeter H, et al. Improving combined diabetes outcomes by adding a simple patient intervention to physician feedback: a cluster randomized trial. The Israel Medical Association journal: IMAJ. 2009;11(12):719-24.

103. Winickoff Rn Fau - Coltin KL, Coltin Kl Fau - Morgan MM, Morgan Mm Fau - Buxbaum RC, Buxbaum Rc Fau - Barnett GO, Barnett GO. Improving physician performance through peer comparison feedback. Medical care. 1984:527-34.

104. Winslade N, Eguale T, Tamblyn R. Optimising the changing role of the community pharmacist: a randomised trial of the impact of audit and feedback. BMJ Open. 2016;6(5):e010865.

105. Wright FC, Law CHL, Last LD, Klar N, Ryan DP, Smith AJ. A blended knowledge translation initiative to improve colorectal cancer staging [ISRCTN56824239]. BMC Health Services Research. 2006;6(1):4.

106. Young JM, D'Este C Fau - Ward JE, Ward JE. Improving family physicians' use of evidence-based smoking cessation strategies: a cluster randomization trial. Preventive medicine. 2002;35(6):572-83.

**Appendix 6 – Study and intervention characteristics of each included comparison (n=117)**

|  | STUDY CHARACTERISTICS | | | | | INTERVENTION CHARACTERISTICS | | | | | | |
| --- | --- | --- | --- | --- | --- | --- | --- | --- | --- | --- | --- | --- |
| Study | **Country** | **Setting** | **Type of Health Worker** | **Target Behaviour** | **Type of Trial** | **Format** | **Frequency** | **Source** | **Internal/ External Delivery** | **Ref Group** | **BCTs** | **Direction of Change** |
| Aspy 2008 | USA | Primary | Doctor GP | Tests | Cluster RCT | Mixed | More than twice | Investigator | External | Peer | SC & other BCTs | Increase |
| Awad 2006 A | Other/ Multiple | Primary | Mixed or team | Prescribing | RCT | Unclear/ Not reported | Only once | Unclear/ Not reported | Unclear/ Not reported | Peer | SC | Increase |
| Awad 2006 B | Other/ Multiple | Primary | Mixed or team | Prescribing | RCT | Unclear/ Not reported | Only once | Unclear/ Not reported | Unclear/ Not reported | Peer | SC & other BCTs | Increase |
| Baker 1997 | UK | Primary | Doctor GP | Management/ communication re condition | Cluster RCT | Unclear/ Not reported | Only once | Unclear/ Not reported | Unclear/ Not reported | Peer | SN both arms | Increase |
| Barnett 2014 Arm A | UK | Primary | Doctor GP | Prescribing | Cluster RCT | Email | More than twice | Investigator | Unclear/ Not reported | Multiple | SC & CS | Increase |
| Barnett 2014 Arm B | UK | Primary | Doctor GP | Prescribing | Cluster RCT | Email | More than twice | Investigator | Unclear/ Not reported | Multiple | multiple SN & other BCTs | Increase |
| Baskerville 2001 | Canada | Primary | Doctor GP | Management/ communication re condition | RCT | Mixed | Unclear/ Not reported | Unclear/ Not reported | Unclear/ Not reported | Unclear/ Not reported | CS & other BCTs | Increase |
| Beck 2005 | Canada | Hospital | Other HCP | Prescribing | Cluster RCT | Written | Only once | Investigator | Unclear/ Not reported | Unclear/ Not reported | SC | Increase |
| Beidas 2017 | USA | Community | Other HCP | Prescribing | RCT | Email | Only once | Supervisor or senior colleague | Internal | Senior Person | SR | Increase |
| Bentz 2007 | USA | Primary | Mixed or team | Management/ communication re condition | Cluster RCT | Written | More than twice | Investigator | Internal | Peer | SC | Increase |
| Bhatia 2017 | Other/ Multiple | Mixed | Mixed or team | Tests | Cluster RCT | Mixed | More than twice | Investigator | External | Peer | SC | Decrease |
| Bhattacharyya 2010 | Canada | Mixed | Mixed or team | Prescribing | Cluster RCT | Unclear/ Not reported | Unclear/ Not reported | Investigator | Internal | Peer | SC | Increase |
| Billue 2012 | USA | Primary | Doctor GP | Prescribing | Cluster RCT | Separate computerised | Twice | Investigator | External | Peer | SC & other BCTs | Increase |
| Boet 2018 | Canada | Hospital | Doctor Secondary care | Management/ communication re condition | Cluster RCT | Email | More than twice | Investigator | External | Peer | SC | Increase |
| Braybrook 1996 | UK | Primary | Doctor GP | Prescribing | RCT | Mixed | Only once | Investigator | External | Professional Body | SN both arms | Decrease |
| Brinkman 2007 | USA | Hospital | Doctor Secondary care | Management/ communication re condition | RCT | Unclear/ Not reported | Unclear/ Not reported | Investigator | External | Multiple | multiple SN & other BCTs | Increase |
| Brown 2018 | Australia | Hospital | Doctor Secondary care | Referrals | Stepped wedge | Mixed | Only once | *Credible source* | Internal | Senior Person | SC & CS & other BCTs | Increase |
| Buntix 1993 | Other/ Multiple | Mixed | Mixed or team | Tests | RCT | Unclear/ Not reported | Only once | Investigator | External | Peer | SC | Increase |
| BUTLER 2012 | UK | Primary | Mixed or team | Prescribing | Cluster RCT | Face to face | Only once | Investigator | External | Peer | SC & other BCTs | Decrease |
| Canovas 2009 | Other/ Multiple | Primary | Doctor GP | Multiple | RCT | Unclear/ Not reported | Unclear/ Not reported | Investigator | External | Peer | SC | Increase |
| Carney 2012 | USA | Hospital | Other HCP | Tests | RCT | Separate computerised | Unclear/ Not reported | Investigator | External | Peer | SC & other BCTs | Decrease |
| Cheater 2006 A | UK | Primary | Other HCP | Multiple | Cluster RCT | Written | Unclear/ Not reported | Investigator | External | Peer | SC | Increase |
| Cheater 2007 B | UK | Primary | Other HCP | Multiple | Cluster RCT | Written | Unclear/ Not reported | Investigator | External | Peer | SC | Increase |
| Colon-Emeric 2007 | USA | Care/ Nursing home | Mixed or team | Prescribing | Cluster RCT | Written | More than twice | Investigator | Unclear/ Not reported | Peer | SC & social support | Increase |
| Curtis 2005 | USA | Other | Doctor GP | Prescribing | RCT | Written | Twice | Investigator | External | Peer | SC | Increase |
| Curtis 2007 | USA | Mixed | Mixed or team | Multiple | Cluster RCT | Separate computerised | Unclear/ Not reported | Investigator | External | Peer | SC | Increase |
| Desveaux 2016 | Canada | Care/ Nursing home | Mixed or team | Prescribing | Cluster RCT | Separate computerised | More than twice | Investigator | External | Peer | SN both arms | Decrease |
| Eccles 2000 A | UK | Mixed | Doctor GP | Tests | Factorial | Unclear/ Not reported | Twice | Investigator | External | Peer | SC | Decrease |
| Eccles 2001 B | UK | Mixed | Doctor GP | Tests | Factorial | Unclear/ Not reported | Twice | Investigator | External | Peer | SC | Decrease |
| Elouafkaoui 2016 | UK | Community | Other HCP | Prescribing | Cluster RCT | Unclear/ Not reported | More than twice | Investigator | External | Peer | SC | Decrease |
| Eltayeb 2005 | Other/ Multiple | Primary | Mixed or team | Prescribing | RCT | Unclear/ Not reported | More than twice | Investigator | External | Peer | SC | Decrease |
| FergusonJr 2003 | USA | Hospital | Doctor Secondary care | Prescribing | Cluster RCT | Unclear/ Not reported | Unclear/ Not reported | Investigator | External | Peer | SC | Increase |
| Fiks 2017 | USA | Primary | Doctor GP | Management/ communication re condition | Cluster RCT | Mixed | Twice | Investigator | External | Peer | SC & social support | Increase |
| Foster 2007 | UK | Primary | Doctor GP | Management/ communication re condition | RCT | Written | Only once | Investigator | External | Peer | SC & other BCTs | Increase |
| Foy 2004 | UK | Hospital | Doctor Secondary care | Referrals | Matched pairs cluster RCT | Unclear/ Not reported | Unclear/ Not reported | Investigator | External | Peer | SC & other BCTs | Increase |
| French 2013 | Australia | Primary | Doctor GP | Tests | Cluster RCT | Mixed | Unclear/ Not reported | *Credible source* | Internal | Peer | CS & other BCTs | Decrease |
| Fuller 2012 | UK | Hospital | Mixed or team | Handwashing/ hygiene | Stepped wedge | Mixed | More than twice | Investigator | External | Unclear/ Not reported | SR & other BCTs | Increase |
| Gerber 2013 | USA | Primary | Doctor GP | Prescribing | Cluster RCT | Mixed | More than twice | Investigator | External | Peer | SC & other BCTs | Decrease |
| Gjelstad 2006 | Other/ Multiple | Primary | Doctor GP | Prescribing | Cluster RCT | Written | Unclear/ Not reported | Investigator | Internal | Peer | SC & social support | Decrease |
| Goff 2002 | USA | Primary | Doctor GP | Prescribing | Cluster RCT | Unclear/ Not reported | More than twice | Unclear/ Not reported | Unclear/ Not reported | Peer | SC & instructions & prompts/ cues | Increase |
| Guadagnoli 2000 | USA | Hospital | Doctor Secondary care | Management/ communication re condition | Cluster RCT | Face-to-face | Unclear/ Not reported | *Credible source* | Internal | Peer | CS | Increase |
| Guldberg 2011 | Denmark | Primary | Doctor GP | Multiple | Cluster RCT | Separate computerised | More than twice | Investigator | External | Peer | SC & prompts/ cues | Increase |
| Hallsworth 2016 | UK | Primary | Doctor GP | Prescribing | RCT | Written | Only once | *Credible source* | External | Peer | SC & other BCTs | Decrease |
| Hayashino 2016 | Other/ Multiple | Primary | Doctor GP | Multiple | Cluster RCT | Written | More than twice | Investigator | External | Peer | SC | Increase |
| Hayes 2002 | USA | Hospital | Doctor Secondary care | Management/ communication re condition | Cluster RCT | Face-to-face | Unclear/ Not reported | *Credible source* | Unclear/ Not reported | Unclear/ Not reported | CS | Increase |
| Heller 2001 | Australia | Hospital | Mixed or team | Multiple | Cluster RCT | Mixed | Unclear/ Not reported | *Credible source* | Unclear/ Not reported | Multiple | SC | Unclear |
| Hemkens 2017 | Other/ Multiple | Primary | Doctor GP | Prescribing | RCT | Mixed | More than twice | Investigator | External | Peer | SC | Decrease |
| Herbert 2004 | Canada | Primary | Doctor GP | Prescribing | Cluster RCT | Written | Unclear/ Not reported | Investigator | External | Peer | SC | Increase |
| Horbar 2004 | USA | Hospital | Mixed or team | Prescribing | Cluster RCT | Unclear/ Not reported | Unclear/ Not reported | Investigator | External | Peer | SC & other BCTs | Increase |
| Houston 2015 | USA | Primary | Mixed or team | Referrals | Cluster RCT | Separate computerised | Unclear/ Not reported | Investigator | External | Peer | SC & other BCTs | Increase |
| Howe 1996 | UK | Primary | Doctor GP | Management/ communication re condition | RCT | Unclear/ Not reported | Only once | Investigator | External | Peer | SC & other BCTs | Increase |
| Huis 2013 | Netherlands | Hospital | Other HCP | Handwashing/ hygiene | Cluster RCT | Unclear/ Not reported | Twice | Investigator | External | Peer | SN both arms | Increase |
| Hysong 2012 | USA | Primary | Mixed or team | Management/ communication re condition | Factorial | Separate computerised | More than twice | Investigator | External | Peer | SN both arms | Increase |
| Ivers 2003 | Canada | Primary | Doctor GP | Multiple | Cluster RCT | Written | More than twice | Investigator | External | Peer | SN both arms | Increase |
| Katz 2004 | USA | Primary | Mixed or team | Management/ communication re condition | Cluster RCT | Unclear/ Not reported | Only once | Investigator | External | Peer | SC & instructions & prompts/ cues | Increase |
| Kaufman 2016 | USA | Hospital | Doctor Secondary care | Prescribing | Cluster RCT | Email | More than twice | Investigator | Unclear/ Not reported | Peer | SC | Decrease |
| Kennedy 2015 | Canada | Care/ Nursing home | Mixed or team | Prescribing | Cluster RCT | Unclear/ Not reported | Unclear/ Not reported | Investigator | External | Peer | SC & CS & other BCTs | Increase |
| Kiefe 2001 | USA | Other | Mixed or team | Prescribing | RCT | Written | Unclear/ Not reported | Investigator | External | Peer | SN both arms | Increase |
| Kim 1999 | USA | Primary | Doctor GP | Multiple | Cluster RCT | Written | Only once | Other | Unclear/ Not reported | Peer | SC & social support | Increase |
| Koff 2016 | USA | Hospital | Mixed or team | Handwashing/ hygiene | RCT | Email | More than twice | Investigator | Unclear/ Not reported | Peer | SN both arms | Increase |
| Kogan 2003 | USA | Mixed | Mixed or team | Management/ communication re condition | Cluster RCT | Written | Only once | Supervisor or senior colleague | Internal | Peer | SC | Increase |
| Lakshminarayan 2010 | USA | Hospital | Mixed or team | Management/ communication re condition | Cluster RCT | Unclear/ Not reported | Unclear/ Not reported | *Credible source* | Internal | Senior Person | CS & other BCTs | Increase |
| Leviton 1999 | USA | Hospital | Doctor Secondary care | Prescribing | Cluster RCT | Face/-to-face | Only once | *Credible source* | External | Senior Person | CS & other BCTs | Increase |
| Liddy 2011 | Canada | Primary | Doctor GP | Multiple | Stepped wedge | Face-to-face | Unclear/ Not reported | Investigator | External | Peer | SC & other BCTs | Increase |
| Lim 2018 | Other/ Multiple | Primary | Doctor GP | Prescribing | Cluster RCT | Written | More than twice | Investigator | External | Peer | SC | Decrease |
| Lomas 1991 | Canada | Hospital | Doctor Secondary care | Management/ communication re condition | Cluster RCT | Mixed | More than twice | Peer | Internal | Unclear/ Not reported | CS | Unclear |
| MacLean 2009 | USA | Primary | Mixed or team | Tests | Cluster RCT | Written | More than twice | Investigator | Internal | Peer | SC & prompts/ cues | Increase |
| Mayne 2014 A | USA | Primary | Mixed or team | Prescribing | Cluster RCT | Unclear/ Not reported | More than twice | Investigator | External | Peer | SC & other BCTs | Increase |
| Mayne 2014 B | USA | Primary | Mixed or team | Prescribing | Cluster RCT | Unclear/ Not reported | More than twice | Investigator | External | Peer | SC & other BCTs | Increase |
| McClellan 2003 | Australia | Community | Mixed or team | Management/ communication re condition | Cluster RCT | Mixed | Unclear/ Not reported | *Credible source* | External | Peer | SC & CS | Increase |
| McCluskey 2016 | USA | Primary | Doctor GP | Tests | Cluster RCT | Face-to-face | Only once | Investigator | External | Peer | SC & instructions & prompts/ cues | Increase |
| McPhee 1989 | USA | Hospital | Doctor Secondary care | Tests | RCT | Face-to-face | More than twice | Investigator | External | Peer | SC & information on health consequences | Increase |
| Meeker 2016 | USA | Primary | Doctor GP | Prescribing | Cluster RCT | Email | More than twice | Investigator | External | Peer | SC&SR | Decrease |
| Metlay 2007 | USA | Hospital | Doctor Secondary care | Prescribing | Cluster RCT | Unclear/ Not reported | Only once | Investigator | External | Peer | SC & information on health consequences | Decrease |
| Mold 2008 | USA | Primary | Mixed or team | Management/ communication re condition | RCT | Unclear/ Not reported | Only once | Investigator | External | Peer | SN both arms | Increase |
| Mold 2014 A | USA | Primary | Doctor GP | Management/ communication re condition | Cluster RCT | Face-to-face | More than twice | Peer | Internal | Peer | SC | Increase |
| Mold 2014 B | USA | Primary | Doctor GP | Management/ communication re condition | Cluster RCT | Face-to-face | More than twice | Peer | Internal | Peer | SC | Increase |
| Morrison 2015 | Canada | Hospital | Mixed or team | Management/ communication re condition | Stepped wedge | Unclear/ Not reported | More than twice | Unclear/ Not reported | Unclear/ Not reported | Peer | SC & other BCTs | Increase |
| Mourad 2011 | Netherlands | Other | Mixed or team | Multiple | Cluster RCT | Mixed | Twice | Investigator | External | Peer | SN both arms | Increase |
| O'Connell 1999 | Australia | Primary | Doctor GP | Prescribing | Cluster RCT | Written | Twice | Investigator | External | Peer | SC | Decrease |
| O'Connor 2009 A | USA | Primary | Doctor GP | Tests | RCT | Unclear/ Not reported | More than twice | Investigator | External | Peer | SC & instruction on how to perform the behaviour & prompts/cues | Increase |
| O'Connor 2009 B | USA | Primary | Doctor GP | Tests | RCT | Unclear/Not reported | More than twice | Investigator | External | Peer | SC & instructions & prompts/cues | Increase |
| Patel 2018 | USA | Primary | Doctor GP | Prescribing | Cluster RCT | Unclear/ Not reported | Unclear/ Not reported | Investigator | External | Peer | SC | Increase |
| Peiris 2015 | Australia | Primary | Mixed or team | Tests | Cluster RCT | Unclear/ Not reported | Unclear/ Not reported | Investigator | External | Peer | SC & prompts/cues | Increase |
| Persell 2016 | USA | Primary | Doctor GP | Prescribing | Factorial | Email | More than twice | Investigator | External | Peer | SC&SR | Decrease |
| Pimlott 2003 | Canada | Primary | Doctor GP | Prescribing | RCT | Written | More than twice | Investigator | External | Peer | SC & information on health consequences | Decrease |
| Pope 2010 | Canada | Hospital | Doctor Secondary care | Other | RCT | Face-to-face | Only once | Investigator | External | Peer | SC & social support (unspecified | Increase |
| Quinley 2004 | USA | Primary | Doctor GP | Prescribing | Cluster RCT | Written | Only once | Investigator | External | Peer | SN both arms | Increase |
| Raasch 2000 | Australia | Primary | Doctor GP | Tests | RCT | Mixed | Only once | Peer | External | Peer | SC & social support (unspecified) | Increase |
| Raja 2015 | USA | Hospital | Doctor Secondary care | Tests | RCT | Email | More than twice | Unclear/ Not reported | Internal | Peer | SC & prompts/cues | Increase |
| Rask 2001 | USA | Primary | Mixed or team | Tests | Cluster RCT | Written | Only once | *Credible source* | External | Senior Person | SN both arms | Increase |
| Sandbaek 1999 | Denmark | Primary | Doctor GP | Management/ communication re condition | RCT | Written | Unclear/ Not reported | Investigator | External | Peer | SC & other BCTs | Increase |
| Sauaia 2000 | USA | Hospital | Mixed or team | Management/ communication re condition | Cluster RCT | Mixed | Twice | *Credible source* | Unclear/ Not reported | Peer | SN both arms | Increase |
| Schneider 2008 | Other/ Multiple | Primary | Doctor GP | Management/ communication re condition | Cluster RCT | Unclear/ Not reported | Unclear/ Not reported | Unclear/ Not reported | Unclear/ Not reported | Peer | SN both arms | Increase |
| Soleynami | Other/ Multiple | Mixed | Mixed or team | Prescribing | RCT | Written | Only once | Investigator | External | Peer | SC & other BCTs | Decrease |
| Sondergaard 2002 B | Denmark | Primary | Doctor GP | Prescribing | Cluster RCT | Written | More than twice | Investigator | External | Peer | SC | Increase |
| Sondergaard 2003 | Denmark | Primary | Doctor GP | Prescribing | Cluster RCT | Written | Only once | Investigator | External | Peer | SC | Decrease |
| Soumerai 1998 | USA | Hospital | Doctor Secondary care | Prescribing | Cluster RCT | Face to face | Only once | *Credible source* | External | Senior Person | SN both arms | Increase |
| Stewardson 2016 A | Other/ Multiple | Hospital | Mixed or team | Handwashing/ hygiene | Cluster RCT | Face to face | More than twice | *Credible source* | Internal | Senior Person | SC & CS & other BCTs | Increase |
| Stewardson 2016 B | Other/ Multiple | Hospital | Mixed or team | Handwashing/ hygiene | Cluster RCT | Face to face | More than twice | Patient | External | Patient(s) | multiple SN & other BCTs | Increase |
| Thomas 2006 | UK | Primary | Doctor GP | Tests | Factorial | Written | More than twice | Investigator | External | Peer | SC | Decrease |
| TJIA 2015 B | USA | Care/ Nursing home | Mixed or team | Prescribing | Cluster RCT | Unclear/ Not reported | Only once | Investigator | External | Peer | SC & other BCTs | Decrease |
| TJIA 2015 A | USA | Care/ Nursing home | Mixed or team | Prescribing | Cluster RCT | Unclear/ Not reported | Only once | Investigator | External | Peer | SC & information on health consequences | Decrease |
| Trietsch 2017 | Netherlands | Primary | Doctor GP | Multiple | Cluster RCT | Face to face | More than twice | Investigator | External | Peer | SC & other BCTs | Decrease |
| VanBruggen 2008 | Netherlands | Primary | Mixed or team | Tests | Cluster RCT | Unclear/ Not reported | Only once | Unclear/ Not reported | Unclear/ Not reported | Peer | SC & other BCTs | Increase |
| Vellinga 2016 Arm A | Other/ Multiple | Primary | Doctor GP | Prescribing | Cluster RCT | Separate computerised | Only once | Investigator | External | Peer | SC & prompts/ cues | Increase |
| Vellinga 2016 Arm B | Other/ Multiple | Primary | Doctor GP | Prescribing | Cluster RCT | Separate computerised | Only once | Investigator | External | Peer | SC & other BCTs | Increase |
| Verstappen 2003 A | Netherlands | Primary | Doctor GP | Tests | RCT | Mixed | More than twice | *Credible source* | External | Senior Person | SC & CS & other BCTs | Decrease |
| Verstappen 2003 B | Netherlands | Primary | Doctor GP | Tests | RCT | Written | More than twice | Investigator | External | Peer | SC | Decrease |
| Voorn 2017 | Netherlands | Hospital | Doctor Secondary care | Other | Cluster RCT | Email | Twice | Investigator | External | Peer | SC & other BCTs | Decrease |
| Wang 2018 | Other/ Multiple | Hospital | Mixed or team | Management/ communication re condition | Cluster RCT | Separate computerised | More than twice | Peer | Internal | Peer | SC & social support | Increase |
| Watkins 2004 | USA | Hospital | Doctor Secondary care | Tests | RCT | Unclear/ Not reported | Only once | Investigator | External | Peer | SC & other BCTs | Increase |
| Weitzman | Other/ Multiple | Primary | Doctor GP | Tests | Cluster RCT | Face to face | Only once | Investigator | External | Peer | SN both arms | Increase |
| Winickoff 1984 | USA | Hospital | Doctor Secondary care | Tests | Cluster RCT | Written | More than twice | Investigator | Internal | Peer | SC | Increase |
| Winslade 2016 | Canada | Community | Other HCP | Management/ communication re condition | RCT | Written | Only once | Investigator | External | Peer | multiple SN & other BCTs | Increase |
| Wright 2006 | Canada | Hospital | Doctor Secondary care | Tests | Cluster RCT | Mixed | More than twice | *Credible source* | External | Senior Person | SN both arms | Increase |
| Young 2002 | Australia | Primary | Doctor GP | Management/ communication re condition | Cluster RCT | Face to face | Only once | Peer | Unclear/ Not reported | Peer | SC & other BCTs | Increase |
| *Notes: BCT = behaviour change technique; GP = general practitioner; RCT = randomised controlled trial; SC = social comparison; SN = social norms; CS = credible source; HCP = health care professional; SR = social reward* | | | | | | | | | | | | |

**Appendix 7 – Sensitivity Analyses**

**Sensitivity analysis for overall result, fixed effects**

| **Analysis** | **Effect (95%CI)** | **n** |
| --- | --- | --- |
| ***Full data set*** | **0.08 (0.07 to 0.10)** | **100** |
| Using imputed ICC = 0.2 instead of 0.1 | 0.08 (0.06 to 0.10) | 100 |
| Using imputed ICC = 0.05 instead of 0.1 | 0.09 (0.07 to 0.10) | 100 |
| Removing trials with imputed SDs | 0.09 (0.07 to 0.10) | 94 |
| Removing trials reporting mean % compliance close to 0% or 100%# | 0.07 (0.05 to 0.08) | 77 |
| Keeping only trials at low risk of bias due to allocation concealment | 0.11 (0.09 to 0.13) | 72 |
| Keeping only trials at low risk of bias due to sequence generation | 0.08 (0.06 to 0.10) | 74 |
| Keeping only trials at low risk of bias due to selective outcome reporting | 0.09 (0.07 to 0.11) | 41 |
| Keeping only trials at low risk of bias due to attrition | 0.10 (0.08 to 0.12) | 57 |
| Keeping only trials at low risk of bias due to other biases | 0.09 (0.07 to 0.11) | 59 |
| Removing trials where ‘Feedback on desired behaviour’ not part of tested intervention | 0.08 (0.07 to 0.10) | 88 |

#Trials using mean % compliance and reporting mean % compliance below 20% or above 80%

**Sensitivity analysis for overall result, random effects**

| **Analysis** | **Effect (95%CI)** | **n** |
| --- | --- | --- |
| ***Full data set*** | **0.16(0.11 to 0.22)** | **100** |
| Using imputed ICC = 0.2 instead of 0.1 | 0.16(0.10 to 0.21) | 100 |
| Using imputed ICC = 0.05 instead of 0.1 | 0.16(0.11 to 0.21) | 100 |
| Removing trials with imputed SDs | 0.18(0.12 to 0.23) | 94 |
| Removing trials reporting mean % compliance close to 0% or 100%# | 0.12(0.07 to 0.16) | 77 |
| Keeping only trials at low risk of bias due to allocation concealment | 0.18(0.12 to 0.25) | 72 |
| Keeping only trials at low risk of bias due to sequence generation | 0.17(0.10 to 0.23) | 74 |
| Keeping only trials at low risk of bias due to selective outcome reporting | 0.22(0.13 to 0.31) | 41 |
| Keeping only trials at low risk of bias due to attrition | 0.18(0.13 to 0.24) | 57 |
| Keeping only trials at low risk of bias due to other biases | 0.13(0.09 to 0.18) | 59 |
| Removing trials where ‘Feedback on desired behaviour’ not part of tested intervention | 0.17(0.12 to 0.23) | 88 |

#Trials using mean % compliance and reporting mean % compliance below 20% or above 80%
